# Dietary Protein Sources and Incidence of Breast Cancer: A Dose-Response Meta-Analysis of Prospective Studies

**DOI:** 10.3390/nu8110730

**Published:** 2016-11-17

**Authors:** Jing Wu, Rong Zeng, Junpeng Huang, Xufeng Li, Jiren Zhang, James Chung-Man Ho, Yanfang Zheng

**Affiliations:** 1 Oncology Center, Zhujiang Hospital of Southern Medical University, Guangzhou 510000, China; m15521144080@163.com (J.W.); zengrongtannan@163.com (R.Z.); fjhuangjunpeng@163.com (J.H.); 15018731689@163.com (X.L.); drjirenzhang@163.com (J.Z.); 2 Division of Respiratory Medicine, Department of Medicine, The University of Hong Kong, Queen Mary Hospital, Hong Kong, China; jhcom@hku.hk

**Keywords:** breast cancer, dietary protein sources, prospective studies, meta-analysis

## Abstract

Protein is important to the human body, and different sources of protein may have different effects on the risk of breast cancer. Thus, we conducted a meta-analysis to investigate the association between different dietary protein sources and breast cancer risk. PubMed and several databases were searched until December 2015. Relevant articles were retrieved according to specific searching criteria. Forty-six prospective studies were included. The summary relative risk (RR) for highest versus lowest intake was 1.07 (95% confidence interval (CI) 1.01–1.14, *I*^2^ = 34.6%) for processed meat, 0.92 (95% CI 0.84–1.00, *I*^2^ = 0%) for soy food, 0.93 (95% CI 0.85–1.00, *I*^2^ = 40.1%) for skim milk, and 0.90 (95% CI 0.82–1.00, *I*^2^ = 0%) for yogurt. Similar conclusions were obtained in dose-response association for each serving increase: total red meat (RR: 1.07; 95% CI 1.01–1.14, *I*^2^ = 7.1%), fresh red meat (RR: 1.13; 95% CI 1.01–1.26, *I*^2^ = 56.4%), processed meat (RR: 1.09; 95% CI 1.02–1.17, *I*^2^ = 11.8%), soy food (RR: 0.91; 95% CI 0.84–1.00, *I*^2^ = 0%), and skim milk (RR: 0.96; 95% CI 0.92–1.00, *I*^2^ = 11.9%). There was a null association between poultry, fish, egg, nuts, total milk, and whole milk intake and breast cancer risk. Higher total red meat, fresh red meat, and processed meat intake may be risk factors for breast cancer, whereas higher soy food and skim milk intake may reduce the risk of breast cancer.

## 1. Introduction

Protein is important to the human body. Protein is involved in the constitution of human tissues and the regulation of various physiological functions. It is essential for body growth and development, as well as the transport of many important substances, and the provision of biological energy. Protein deficiency can induce many types of diseases, such as retarded growth and development, fatigue, and nutritional edema, and it can even be life threatening [1,2].

No food contains exclusively protein; we must always intake different sources of protein from a variety of compounds. Different sources of protein might have different effects on breast cancer risk. For example, carcinogenic byproducts—such as heterocyclic amines and polycyclic aromatic hydrocarbons formed during the high-temperature cooking of red meat [3,4]—can increase the risk of breast cancer. However, evidence from prospective cohort studies remains controversial [5,6,7,8]. Thus, in this study, we wanted to investigate the association between the consumption of different sources of dietary protein and the risk of breast cancer. We also intend for this article to serve as a reference for further research into the relationship between different sources of dietary protein and breast cancer incidence, and as guidance for the different dietary sources of protein intake.

Meat, egg, soy food, and milk are common and important sources of dietary protein. In this meta-analysis, we explored the effects of total red meat (including fresh red meat and processed meat), fresh red meat, processed meat, poultry, fish, egg, soy food, nuts, total milk, whole milk, skim milk, and yogurt on breast cancer incidence.

## 2. Materials and Methods

### 2.1. Search Strategy and Selection Criteria

This meta-analysis was conducted in adherence to the Preferred Reporting Items for Systematic Review and Meta-analysis statement guidelines [9]. We systematically searched the literature without restriction from the following databases: PubMed, ISI Web of Science, and Cochrane Library. The search time was up to December 2015, and the search terms in Table 1 were employed to retrieve the relevant articles.

Studies were included in the meta-analysis if they met the following criteria: (1) prospective cohort studies, nested case–control studies, or case–cohort studies; (2) the relationship between dietary protein sources consumption and risk of breast cancer was evaluated; (3) the outcome of interest was incidence of breast cancer; (4) the relative risk (RR) with corresponding 95% confidence intervals (CI) of breast cancer was reported. Retrospective studies, cross-sectional studies, correlation studies, studies in animals, and studies in mechanisms were excluded. If the articles were duplicated or from the same study population, only the study with the most recent or the most complete study was included.

### 2.2. Data Extraction

The following data were extracted by two independent researchers: The first author’s name, publication year, country, study population, study design, duration of follow-up, number of cases, cohort size, person-years for the population, age at baseline, menopausal status, type of protein, RR with corresponding 95% CIs for each exposure category, and covariates. Disagreements were resolved by the group discussion of all researchers. We selected the effect estimates with more adjustment factors. The Newcastle–Ottawa scale was used to evaluate the quality of each study [10].

### 2.3. Statistical Analysis

Relative risk was used for risk estimates, and it was estimated by hazard ratios or RR in cohort studies and odds ratios in nested case–control studies. We used log transformed RR and its corresponding 95% CIs from each eligible study for the meta-analysis. First, we determined the summary RR of breast cancer, comparing the highest versus the lowest categories with a random-effects model [11]. Then, we performed a dose-response analysis with the method proposed by Greenland and Longnecker [12] to compute linear trends and 95% CIs from the natural logs of the RRs and CIs across categories of each source of protein intake. When the included studies used different units (such as servings and times), we converted them into grams per day according to standard conversions from the Food Standards Agency [13] and other documents [14,15,16] (for fresh red meat, poultry, and fish, one serving was equivalent to 120 g; for egg and processed meat, one serving was equal to 50 g; for dairy products, the weight of one serving was 200 g; for nuts, it was 28 g). For soy foods and nuts, we used serving/day as units, because most studies used servings as units. A few studies [17,18] used g/1000 kcal as units; we also converted them into grams per day according to the average energy intake of this population.

Heterogeneity among studies was assessed using Cochran’s Q and *I*^2^ statistics, and we considered *p*-values < 0.10 and *I*^2^ > 50% to be indicative of significant heterogeneity [19,20]. Subgroup analysis and meta-regression were conducted to examine sources of study heterogeneity and the influence of potential residual confounding factors. We also conducted sensitivity analysis by removing one study at a time and examining the influence of each individual study on the summary results. Publication bias was assessed with Egger’s test and Begg’s test, with the results considered to indicate publication bias when *p* < 0.05. All statistical analyses were performed using STATA version 12.0 (StataCorp, College Station, TX, USA).

## 3. Results

**Figure 1:**
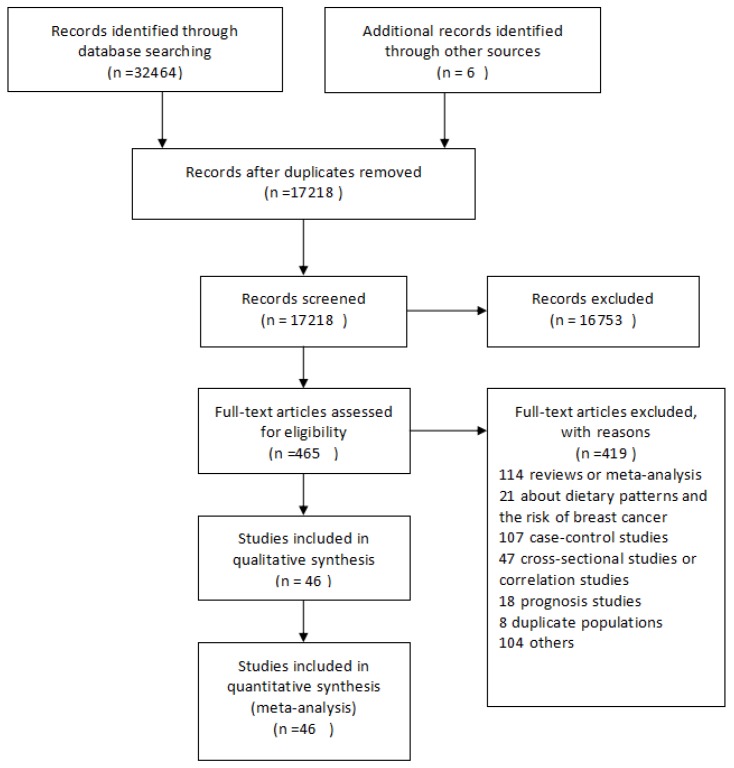
Flow-chart of study selection.

The flowchart of the literature search is presented in Figure 1. In total, forty-six [5,6,7,8,17,18,21,22,23,24,25,26,27,28,29,30,31,32,33,34,35,36,37,38,39,40,41,42,43,44,45,46,47,48,49,50,51,52,53,54,55,56,57,58,59,60] independent prospective cohort studies were identified for meta-analysis. Among these articles, 15 were from the United States [7,8,18,21,24,26,31,34,37,39,40,49,52,55,60], 20 from Europe [5,6,22,23,25,27,29,32,35,38,43,44,46,47,48,50,51,53,54,57], 10 from Asia [17,28,33,36,41,42,45,56,58,59], and one each from North America and Western Europe [30]. The mean follow-up time of 46 eligible articles ranged from 3.9 to 65 years. Among these, four were nested case–control studies [5,24,38,60], one was a case–cohort study [32], and five were secondary analysis of randomized controlled trial data [17,41,44,54,57] (Supplementary Materials Table S1). The average score for the quality assessment of included studies was 7.6, and the scores for all studies were greater than 5 (Supplementary Materials Table S2).

### 3.1. Total Red Meat (Fresh Red Meat and Processed Meats) and Risk of Breast Cancer

Highest versus lowest category analysis. Eight cohort studies [7,8,17,30,34,41,48,58] were included in the analysis, including 19,912 cases among 691,383 participants. The summary RR for highest versus lowest was 1.05 (95% CI 0.95–1.16) with significant heterogeneity, *I*^2^ = 63.1% (See Table 2). No publication bias was observed by Begg’s test (*p* = 0.536) or Egger’s test (*p* = 0.332).

**Figure 2:**
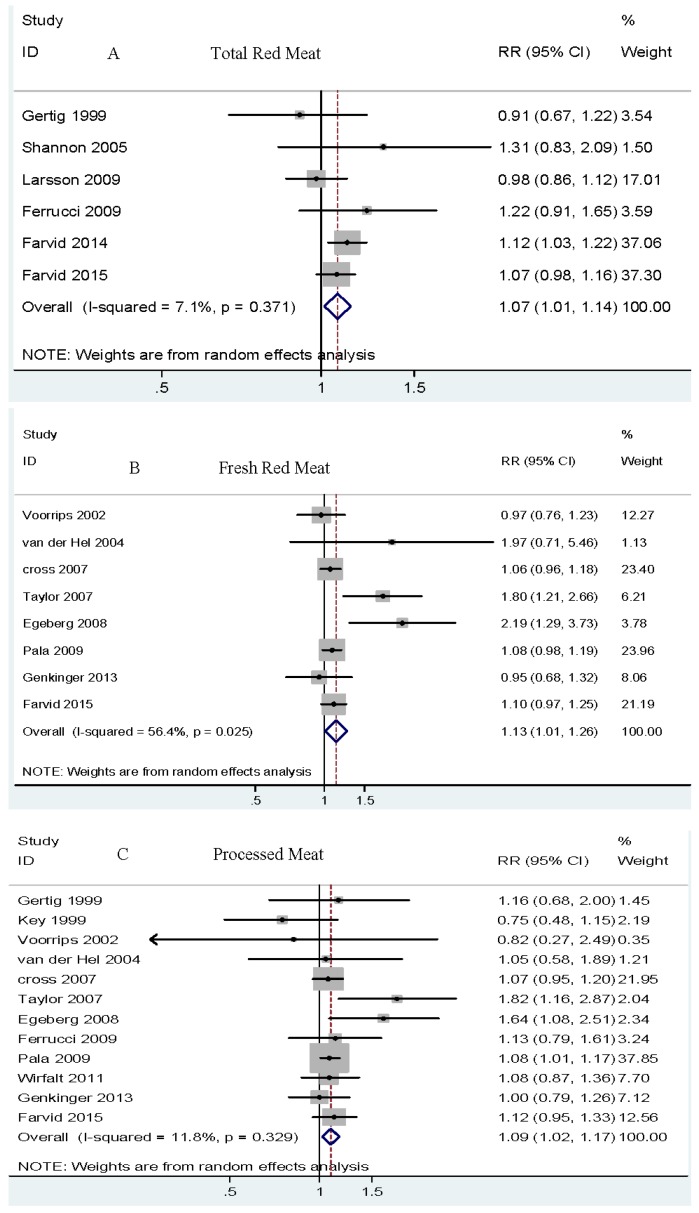

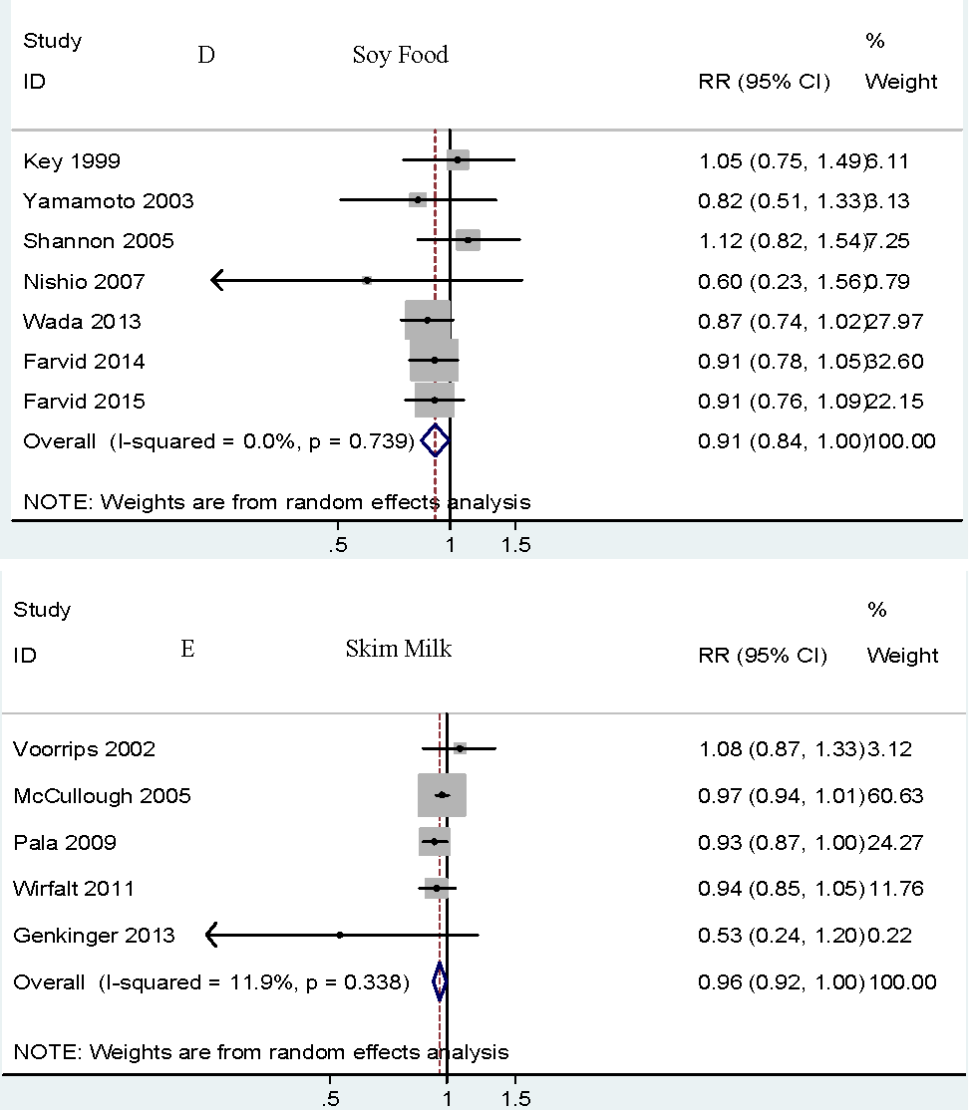
Dose-response analysis of total red meat, fresh red meat, processed meat, soy foods, and skim milk intake and the risk of breast cancer. (**A**) Dose-response analysis of total red meat intake and the risk of breast cancer. (**B**) Dose-response analysis of fresh red meat intake and the risk of breast cancer. (**C**) Dose-response analysis of processed meat intake and the risk of breast cancer. (**D**) Dose-response analysis of soy foods intake and the risk of breast cancer. (**E**) Dose-response analysis of skim milk intake and the risk of breast cancer. Overall, relative risk calculated with random effects model. As shown in the figure, total red meat (A), fresh red meat (B), and processed red meat (C) are related to increased breast cancer risk, whereas soy food (D) and skim milk (E) are related to a decreased risk of breast cancer.

**Figure 3:**
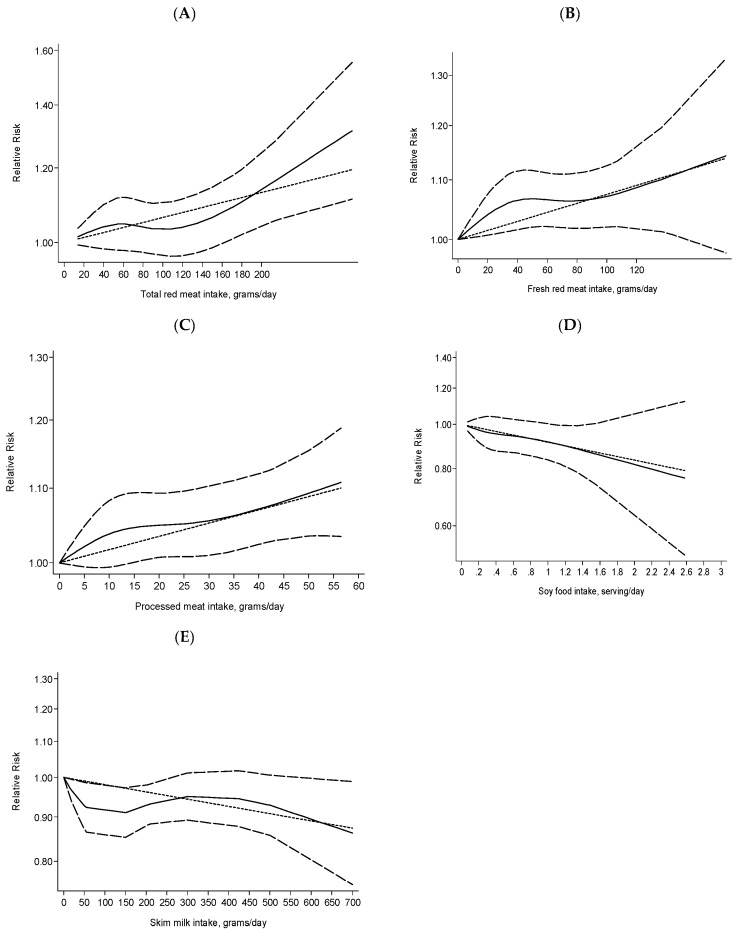
Dose-response analysis of total red meat, fresh red meat, processed meat, soy foods, and skim milk intake and the risk of breast cancer. The solid line represents estimated relative risks (RRs), and dashed lines are their 95% confidence interval (CI). As shown in the figure, total red meat, fresh red meat, and processed red meat are related to an increased risk of breast cancer, whereas soy food and skim milk are related to a decreased risk of breast cancer. (**A**) Total red meat; (**B**) Fresh red meat; (**C**) Processed meat; (**D**) Soy foods; (**E**) Skim milk.

Dose-response analysis. Six cohort studies [7,8,17,41,48,60] were eligible for dose-response analysis. The summary RR per 120 g/day was 1.07 (95% CI 1.01–1.14), with low heterogeneity, *I*^2^ = 7.1% (see Figure 2A and Table 2). No publication bias was observed by Begg’s test (*p* = 1.000) or Egger’s test (*p* = 0.121). We observed a linear association between total red meat intake and increased risk of breast cancer (*p* = 0.157) (see Figure 3A).

### 3.2. Fresh Red Meat and Risk of Breast Cancer

Highest versus lowest category analysis. Twelve cohort studies [5,6,7,18,21,26,32,38,48,50,55,57] were eligible for analysis with 23,667 cases and 1,154,364 participants. The summary RR for highest versus lowest was 1.07 (95% CI 0.98–1.17) with significant heterogeneity, *I*^2^ = 53.3% (See Table 2). No publication bias was observed by Begg’s test (*p* = 0.732) or Egger’s test (*p* = 0.605).

Dose-response analysis. Eight cohort studies [5,6,7,18,32,38,50,55] were included in the dose-response analysis. The summary RR per 120 g/day was 1.13 (95% CI 1.01–1.26) with significant heterogeneity, *I*^2^ = 56.4% (see Figure 2B and Table 2). No publication bias was observed by Begg’s test (*p* = 0.266) or Egger’s test (*p* = 0.110). We observed a linear association between fresh red meat intake and increased breast cancer risk (*p* = 0.292) (see Figure 3B).

### 3.3. Processed Meat and Risk of Breast Cancer

**Figure 4:**
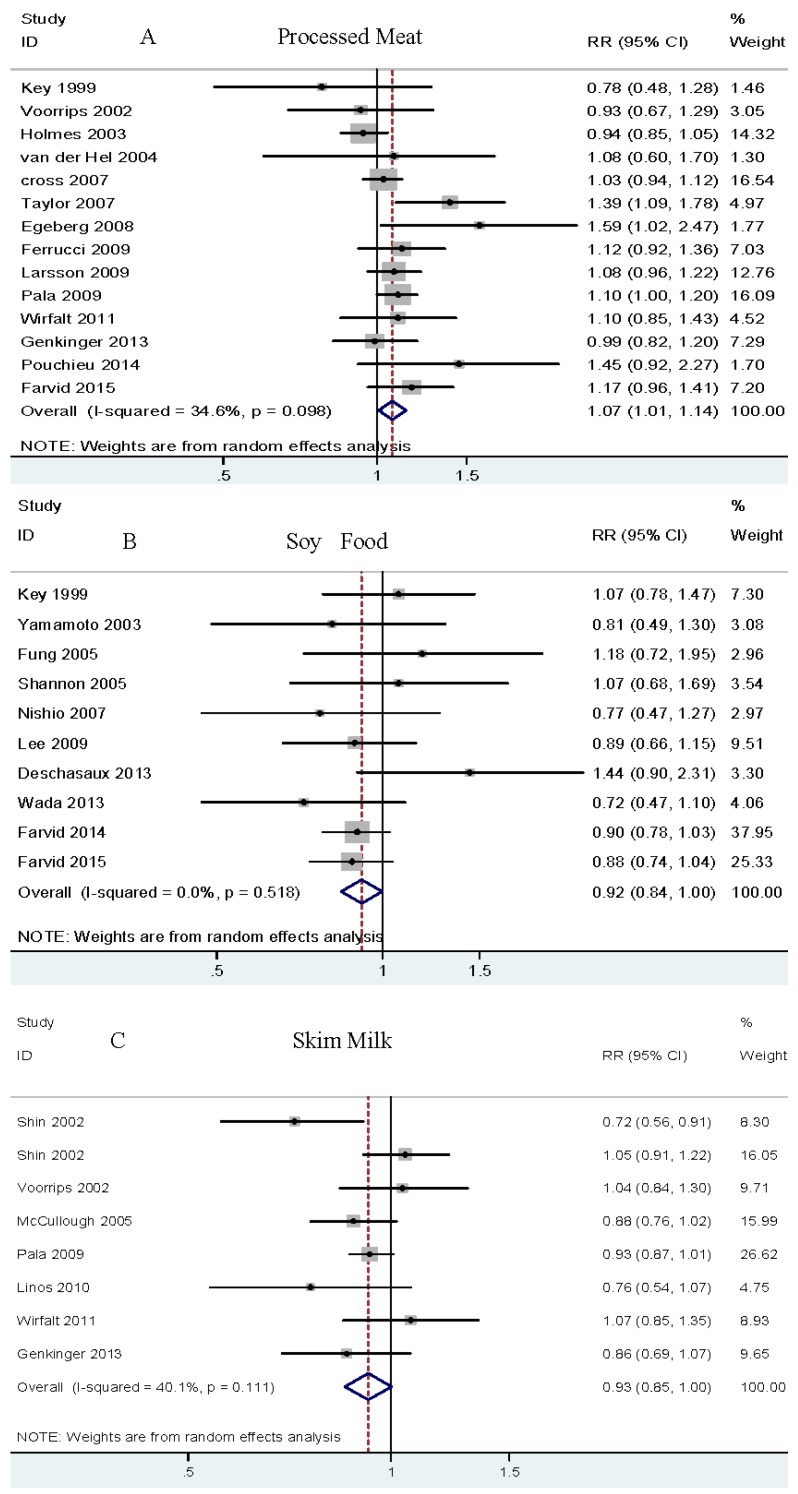

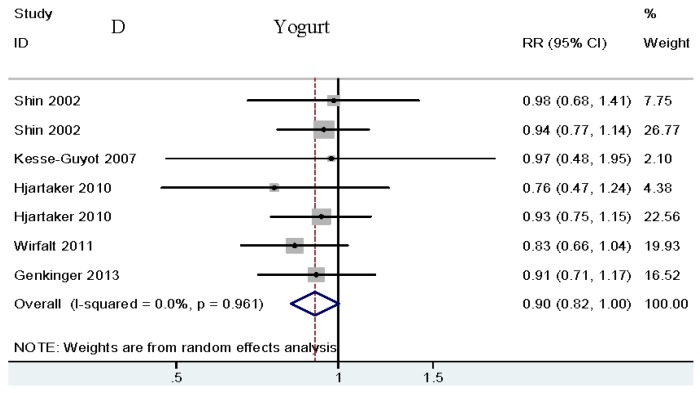
Relative risk of breast cancer for highest vs. lowest category of dietary protein sources intake. (**A**) Relative risk of breast cancer for highest vs. lowest category of processed red meat intake; (**B**) Relative risk of breast cancer for highest vs. lowest category of soy food intake; (**C**) Relative risk of breast cancer for highest vs. lowest category of skim milk intake; (**D**) Relative risk of breast cancer for highest vs. lowest category of yogurt intake. Overall, relative risk calculated with a random effects model. As shown in the figure, processed red meat (**A**) is related to an increased risk of breast cancer, whereas soy food (**B**), skim milk (**C**), and yogurt (**D**) are related to decreased breast cancer risk.

Highest versus lowest category analysis. Fourteen cohort studies [5,6,7,17,18,28,32,34,38,48,50,53,55,57] investigated the association between processed meat intake and breast cancer risk, with 26,952 breast cancer events and 1,235,085 participants. The summary RR for highest versus lowest was 1.07 (95% CI 1.01–1.14) with moderate heterogeneity, *I*^2^ = 34.6% (see Figure 4A and Table 2). No publication bias was found by Begg’s test (*p* = 0.743) or Egger’s test (*p* = 0.251).

Dose-response analysis. In the dose-response, twelve cohort studies [5,6,7,17,18,28,32,38,50,53,55,60] were included. The summary RR per 50 g/day was 1.09 (95% CI 1.02–1.17) with low heterogeneity, *I*^2^ = 11.8% (see Figure 2C and Table 2). No publication bias was observed by Begg’s test (*p* = 0.945) or Egger’s test (*p* = 0.566). We observed a linear association between processed meat intake and increased risk of breast cancer (*p* = 0.633) (see Figure 3C).

### 3.4. Poultry and Risk of Breast Cancer

Highest versus lowest category analysis. Eleven cohort studies [5,6,7,8,17,21,24,34,41,50,55] were included in the analysis, including 19,400 cases among 726,947 participants. The summary RR for highest versus lowest was 1.01 (95% CI 0.93–1.11) with significant heterogeneity, *I*^2^ = 58% (See Table 2). No publication bias was observed by Begg’s test (*p* = 0.755) or Egger’s test (*p* = 0.558).

Dose-response analysis. Ten cohort studies [5,6,7,8,17,21,41,50,55,60] were included in the dose-response analysis. The summary RR per 120 g/day was 0.97 (95% CI 0.85–1.11) with moderate heterogeneity, *I*^2^ = 33.2% (See Table 2). No publication bias was observed by Begg’s test (*p* = 0.107) or Egger’s test (*p* = 0.09). We observed a null association between poultry intake and risk of breast cancer.

### 3.5. Fish and Risk of Breast Cancer

Highest versus lowest category analysis. Eighteen cohort studies [5,7,8,17,21,23,24,28,33,34,35,37,41,42,43,53,55,59] were included in the analysis, including 20,810 cases among 914,451 participants. The summary RR for highest versus lowest was 1.04 (95% CI 0.97–1.12) with moderate heterogeneity, *I*^2^ = 47.9% (See Table 2). No publication bias was observed by Begg’s test (*p* = 0.705) or Egger’s test (*p* = 0.613).

Dose-response analysis. Thirteen cohort studies [5,7,8,17,21,28,37,41,43,53,55,59,60] were eligible for the dose-response analysis. The summary RR per 120 g/day was 1.07 (95% CI 0.94–1.21) with moderate heterogeneity, *I*^2^ = 33.3% (See Table 2). No publication bias was observed by Begg’s test (*p* = 0.100) or Egger’s test (*p* = 0.089). We did not observe a linear association between fish intake and risk of breast cancer.

### 3.6. Egg and Risk of Breast Cancer

Highest versus lowest category analysis. Nine cohort studies [7,8,21,25,28,34,41,50,53] reported the association between egg intake and breast cancer incidence, including 16,910 cases and 639,720 participants. The summary RR for highest versus lowest was 1.04 (95% CI 0.98–1.11) with low heterogeneity, *I*^2^ = 6.7% (See Table 2). No publication bias was observed by Begg’s test (*p* = 0.754) or Egger’s test (*p* = 0.593).

Dose-response analysis. Eight cohort studies [7,8,21,25,28,41,50,53] were eligible for dose-response analysis. The summary RR per 50 g/day was 1.04 (95% CI 0.94–1.15) with moderate heterogeneity, *I*^2^ = 26.9% (See Table 2). No publication bias was observed by Begg’s test (*p* = 0.266) or Egger’s test (*p* = 0.340). We found that egg intake did not influence the risk of breast cancer.

### 3.7. Soy Food and Risk of Breast Cancer

Highest versus lowest category analysis. Ten cohort studies [7,8,28,36,39,41,45,49,54,56] were eligible for analysis, including 12,888 cases among 452,916 participants. The summary RR for highest versus lowest was 0.92 (95% CI 0.84–1.00) with no heterogeneity, *I*^2^ = 0.0% (see Figure 4B and Table 2). No publication bias was observed by Begg’s test (*p* = 0.592) or Egger’s test (*p* = 0.413).

Dose-response analysis. Seven cohort studies [7,8,28,36,41,45,56] were included in the dose-response analysis. The summary RR per serving/day was 0.91 (95% CI 0.84–1.00) with no heterogeneity, *I*^2^ = 0.0% (see Figure 2D and Table 2). No publication bias was observed by Begg’s test (*p* = 0.764) or Egger’s test (*p* = 0.981). We observed a linear association between soy food intake and decreased risk of breast cancer (*p* = 0.908) (see Figure 3D).

### 3.8. Nuts and Risk of Breast Cancer

Highest versus lowest category analysis. Three cohort studies [7,8,47] investigated the association between nut intake and breast cancer risk, including 4506 cases among 148,807 participants. The summary RR for highest versus lowest was 0.96 (95% CI 0.88–1.06), with no heterogeneity, *I*^2^ = 0.0% (See Table 2). No publication bias was observed by Begg’s test (*p* = 1.000) or Egger’s test (*p* = 0.461).

Dose-response analysis. Three cohort studies [7,8,47] were included in the dose-response analysis. The summary RR per serving/day was 0.96 (95% CI 0.84–1.09) with no heterogeneity, *I*^2^ = 0.0% (See Table 2). No publication bias was observed by Begg’s test (*p* = 0.100) or Egger’s test (*p* = 0.955). We did not observe a linear association between nut intake and risk of breast cancer.

### 3.9. Total Milk and Risk of Breast Cancer

Highest versus lowest category analysis. Eighteen studies from sixteen cohorts [22,25,27,28,29,31,32,40,41,44,46,50,51,52,53,55] were eligible for analysis, with 19,747 cases among 775,778 participants. The summary RR for highest versus lowest was 0.92 (95% CI 0.84–1.02) with significant heterogeneity, *I*^2^ = 53.5% (See Table 2). No publication bias was observed by Begg’s test (*p* = 0.495) or Egger’s test (*p* = 0.292).

Dose-response analysis. Eleven cohort studies [25,28,29,32,40,41,44,46,50,53,55] were eligible for the dose-response analysis. The summary RR per 200 g/day was 0.97 (95% CI 0.93–1.01) with moderate heterogeneity, *I*^2^ = 36.4% (See Table 2). No publication bias was observed by Begg’s test (*p* = 0.436) or by Egger’s test (*p* = 0.355). We did not observe a linear association between total milk intake and breast cancer risk.

### 3.10. Whole Milk and Risk of Breast Cancer

Highest versus lowest category analysis. Nine studies from eight cohorts [21,25,26,31,32,50,52,55] were included in analysis, with 13,781 cases among 554,775 participants. The summary RR for highest versus lowest was 0.99 (95% CI 0.87–1.12) with moderate heterogeneity, *I*^2^ = 37.4% (See Table 2). No publication bias was observed by Begg’s test (*p* = 0.917) or Egger’s test (*p* = 0.723).

Dose-response analysis. Five cohort studies [21,25,32,50,55] were eligible for the dose-response analysis. The summary RR per 200 g/day was 1.02 (95% CI 0.92–1.13) with moderate heterogeneity, *I*^2^ = 32.8% (See Table 2). No publication bias was observed by Begg’s test (*p* = 1.000) or Egger’s test (*p* = 0.660). We did not observe a linear association between whole milk intake and risk of breast cancer.

### 3.11. Skim Milk and Risk of Breast Cancer

Highest versus lowest category analysis. Eight studies from seven cohorts [31,32,41,50,52,53,55] reported an association between skim milk intake and risk of breast cancer, including 16,664 cases and 586,726 participants. The summary RR for highest versus lowest was 0.93 (95% CI 0.85–1.00) with moderate heterogeneity, *I*^2^ = 40.1% (see Figure 4C and Table 2). No publication bias was observed by Begg’s test (*p* = 0.266) or Egger’s test (*p* = 0.616).

Dose-response analysis. Five cohort studies [32,41,50,53,55] were eligible for the dose-response analysis. The summary RR per 200 g/day was 0.96 (95% CI 0.92–1.00) with low heterogeneity, *I*^2^ = 11.9%. (see Figure 2E and Table 2). No publication bias was observed by Begg’s test (*p* = 0.806) or Egger’s test (*p* = 0.498). We observed a linear association between skim milk intake and decreased risk of breast cancer (*p* = 0.109) (see Figure 3E).

### 3.12. Yogurt and Risk of Breast Cancer

Highest versus lowest category analysis. Seven studies from five cohorts [31,44,51,53,55] explored an association between yogurt consumption and breast cancer risk, with 6793 cases among 225,057 participants. The summary RR for highest versus lowest was 0.90 (95% CI 0.82–1.00) with no heterogeneity, *I*^2^ = 0.0% (see Figure 4D and Table 2). No publication bias was observed by Begg’s test (*p* = 0.764) or Egger’s test (*p* = 0.77).

Dose-response analysis. Only three cohorts [44,53,55] were eligible for the dose-response analysis. The summary RR per 200 g/day was 0.87 (95% CI 0.72–1.06) with no heterogeneity, *I*^2^ = 0.0% (See Table 2). No publication bias was observed by Begg’s test (*p* = 1.000) or Egger’s test (*p* = 0.488). We did not observe a linear association between yogurt intake and risk of breast cancer.

### 3.13. Subgroup and Sensitivity Analysis

Subgroup analysis and meta-regression were conducted for both highest versus lowest analysis (Supplementary Materials Table S3) and dose-response analysis (Table 3). For most sources of dietary protein exposure, meta-regression and subgroup analysis did not exhibit any substantial change in the summary relative risk. For processed meat and skim milk, the results of subgroup analysis were consistent with summary analysis; however, there was a weak association in some strata (*p* < 0.1). For soy food and total red meat, we did not conduct the subgroup analysis, due to no evidence of heterogeneity in the summary analysis (*I*_soyfood_^2^ = 0.0%, *I*_total red meat_^2^ = 7.1%). Given that only three cohort studies investigated the association between nut and yogurt intake and risk of breast cancer, we also did not conduct the meta-regression and subgroup analysis for nuts and yogurt.

In sensitivity analysis, we removed one study at a time and calculated overall homogeneity and effect size. We confirmed that the summary results were reliable for most sources of dietary protein (data not shown).

## 4. Discussion

This meta-analysis suggested that higher intake of soy food and skim milk could decrease the risk of breast cancer, and that higher intake of processed meat may increase the incidence of breast cancer. Dose-response analysis revealed the summary risk of breast cancer decreased by 9% for soy food, decreased by 4% for skim milk, and increased by 9% for processed meat. For total red meat and fresh red meat, the dose-response analysis suggests that increased intake of these foods could increase the risk of breast cancer, but the results of summary RR for highest versus lowest intake failed to reveal an increased risk. For yogurt, we found it could decrease risk of breast cancer, and the summary RR for highest versus lowest intake was 0.90 (95% CI 0.82–1.00). However, the result was not consistent with the result from dose-response analysis. Only three cohorts were eligible for the dose-response analysis for yogurt. More studies are needed to investigate the association between yogurt intake and risk of breast cancer. We also found a null association between poultry, fish, egg, nut, total milk, and whole milk consumption and risk of breast cancer.

Meat is an important source of protein. NHANES data indicated that approximately 58% of the meat consumed was red meat and that 22% of the total meat consumed was processed meat in the USA [61]. Evidence from prospective studies of red and processed meat consumption and risk of breast cancer was inconsistent [7,8,53,55,57], and the mechanisms involved require further investigation. Several mechanisms were important in explaining the association between red and processed meat intake and risk of breast cancer: (1) carcinogenic byproducts—such as heterocyclic amines and polycyclic aromatic hydrocarbons—formed during high temperature cooking of red meat [4,62]; (2) fat, heme iron, and the animal sugar molecule *N*-glycolyneuraminic acid were enriched in red meat, which could promote inflammation, oxidative stress, and tumor formation [63,64,65]; (3) in a few countries, hormone residues of the exogenous hormones used to stimulate the growth of beef cattle has also been suggested as a risk factor of breast cancer [66].

Soy food is an essential part of many people’s diets in Asian countries. An appropriate method of calculating the amount of protein in soy food is not available, and only one study listed soy protein as an observational item. Thus, we did not extract soy protein from the original data. Soy food contains numerous fibers and phytoestrogens, which can arrest cell cycle, induce apoptosis, and inhibit angiogenesis. These mechanisms might support the notion that soy food intake was negatively associated with breast cancer incidence.

Milk is another important source of protein. Many prospective cohort studies have suggested a null association between total milk consumption and risk of breast cancer [50,51,53,55]. A recent meta-analysis of prospective studies regarding milk consumption and breast cancer incidence found that the intake of total milk was not independently associated with increasing breast cancer risk [64]. They found skim milk could decrease the risk of breast cancer; however, only four studies of skim milk were included in the meta-analysis. Milk contains fat, calcium, vitamin D, conjugated linoleic acids (CLAs), etc. In vitro studies have suggested that calcium, vitamin D, and CLA exert anticarcinogenic effects, such as inhibition of cell cycle progression, induction of apoptosis, inhibition of angiogenesis, and differentiation of mammary cells [67,68,69,70]. However, dietary fat has been thought to be a risk factor of breast cancer [71,72,73]. However, the mechanisms of dietary fat’s influence on cancer risk were speculative, and we need more studies to prove it.

There were few studies that explored the association between yogurt intake and the incidence of breast cancer [31,44,51,53,55]. Most of them hold the idea that yogurt consumption did not impact the risk of breast cancer. However, a study of estrogen- and progesterone receptor-positive (ER+PR+) breast cancer revealed a protective linear risk trend with yogurt (HR = 0.89, 95% CI = 0.80–0.99) [53]. More studies are needed to investigate the association between yogurt intake and risk of breast cancer.

To our knowledge, this is the first meta-analysis to systematically and comprehensively explore the association between different dietary sources of protein and the risk of breast cancer. This is the first meta-analysis to explore the association between poultry, nuts, whole milk, skim milk, and yogurt consumption and breast cancer risk. Additionally, this is an updated meta-analysis to investigate the association between total red meat, fresh red meat, processed meat, fish, egg, soy food, and total milk intake and incidence of breast cancer. Although some previous meta-analyses [15,74,75,76,77] have investigated the association between total red meat, fresh red meat, processed meat, fish, egg, soy food, and total milk intake and incidence of breast cancer, many of them were based on highest versus lowest intake. Since then, a number of large-scale prospective studies were performed. Our study was based on dose-response meta-analysis, and forty-six independent prospective cohort studies were collected. In addition, we also explored the sources of study heterogeneity and the influence of potential residual confounding factors through fifteen subgroup analysis and meta-regression. Recently, a meta-analysis of red meat consumption and breast cancer found that red meat intake was associated with increasing breast cancer risk, but the term “red meat” referred to total red meat in some studies, and fresh red meat in other studies [78]. In our study, we separately explored the association between total red meat (fresh red meat and processed meat), fresh red meat, and processed meat intake and risk of breast cancer.

Our meta-analysis had several strengths. Firstly, the assessment was based on prospective studies, which avoided the influence of recall and selection bias. Secondly, the large sample size (60,615 breast cancer cases and 2,749,307 participants) allowed us to quantitatively assess the association of different sources of protein consumption and breast cancer incidence. Thirdly, our results were based on dose-response meta-analysis, which would avoid some influence of potential residual confounding factors. Finally, we explored the sources of study heterogeneity and the influence of potential residual confounding factors through fifteen subgroup analyses and meta-regression.

However, this meta-analysis also had several limitations. First, the inherent problems of unmeasured or residual confounders in the included studies might confound the association. Although we included fifteen subgroup analyses and meta-regression to explore and balance the influence of potential residual confounding factors (Table 3), numerous unmeasured or residual confounders—such as ER, PR, and Her-2 status of the tumor—might confound the association. Only a few studies [7,8,17,47,48,53,55,59] explored the influence of ER, PR, and Her-2 status of the tumor on the association. In addition, most of the studies used a single Food Frequency Questionnaire and assumed the diet did not change over many years of follow-up. Thus, we compared the studies with different follow-up periods, and the results were consistent (Table 3). However, more high quality studies are needed to investigate the association between yogurt intake and breast cancer risk. Third, different studies used different units (such as servings and times). Although we converted these units into grams per day according to standard conversions from the Food Standards Agency [13] and other documents [14,15,16], this could still influence the association.

## 5. Conclusions

In conclusion, our results suggested that diets rich in soy food and skim milk were associated with decreased breast cancer risk. High intake of red and/or processed meat was associated with an increased risk of breast cancer. A null association was noted between poultry, fish, egg, nut, total milk, and whole milk consumption and risk of breast cancer. However, additional well-designed cohort or interventional studies and studies exploring the mechanisms in humans are needed to confirm the association.

## Figures and Tables

**Table 1:** Search Strategies for meta-analysis.

Topic	Search Strategy
**Breast Cancer**	“Breast Neoplasms” OR “Breast Cancer”
**Study**	“Cohort” OR “Nested Case–control” OR “Case–cohort” OR “Prospective”
**Dietary Protein Sources**	
(1) Red Meat	“Meat” OR “Red Meat” OR “Pork” OR “Beef” OR “Veal” OR “Mutton” OR “Lamb”
(2) Processed Meat	“Meat” OR “Processed Meat” OR “Preserved Meat” OR “Ham” OR “ Sausage” OR “Bacon” OR “Hot Dogs”
(3) Poultry	“Poultry” OR “Chicken” OR “Turkey”
(4) Fish	“Fish” OR “Seafood” OR “Shellfish” OR ” Tuna” OR “Salmon” OR “Sardines” OR “Bluefish”
(5) Egg	“Ovum” OR “Ova” OR “Egg”
(6) Legumes	“Soy” OR “Soy Food” OR “ Soy Protein” OR “ Regular Tofu” OR “ Fried Tofu” OR “Soybeans” OR “String Beans” OR “Beans” OR “Lentils” OR “ Peas” OR “Lima Beans”
(7) Nuts	“Nut” OR “Peanut” OR “Almonds” OR “Pecans” OR “Pistachios” OR “Cashews”
(8) Milks	“Milk” OR “Dietary Products” OR “Whole Milk” OR “Skim Milk” OR “Fermented Milk” OR “Yogurt”

**Table 2:** Associations between specific protein sources and breast cancer incidence.

	High vs. Low		Dose-Response		Serving
	RR	95% CI	RR	95% CI	
Total red meat	1.05	(0.95–1.16)	1.07	(1.01–1.14)	120 g
Fresh red meat	1.07	(0.98–1.17)	1.13	(1.01–1.26)	120 g
Processed meat	1.07	(1.01–1.14)	1.09	(1.02–1.17)	50 g
Poultry	1.01	(0.93–1.11)	0.97	(0.85–1.11)	120 g
Fish	1.04	(0.97–1.12)	1.07	(0.94–1.21)	120 g
Egg	1.04	(0.98–1.11)	1.04	(0.94–1.15)	50 g
Soy food	0.92	(0.84–1.00)	0.91	(0.84–1.00)	Serving
Nuts	0.96	(0.88–1.06)	0.96	(0.84–1.09)	28 g
Total milk	0.92	(0.84–1.02)	0.97	(0.93–1.01)	200 g
Whole milk	0.99	(0.87–1.12)	1.02	(0.92–1.13)	200 g
Skim milk	0.93	(0.85–1.00)	0.96	(0.92–1.00)	200 g
Yogurt	0.90	(0.82–1.00)	0.87	(0.72–1.06)	200 g

**Table 3:** Subgroup analysis of dietary protein sources intake and risk of breast cancer: dose-response meta-analysis.

		**Fresh Red Meat**			**Processed Meat**			**Poultry**			**Fish**		
		***n***	**RR (95% CI)**	***I*^2^**	***n***	**RR (95% CI)**	***I*^2^**	***n***	**RR (95% CI)**	***I*^2^**	***n***	**RR (95% CI)**	***I*^2^**
Overall		8	**1.13 (1.01–1.26)**	56.4	12	**1.09 (1.02–1.17)**	11.8	10	0.97 (0.85–1.11)	33.2	13	1.07 (0.94–1.21)	33.3
Menopausal status:													
Premenopausal		4	1.07 (0.88–1.31)	45.5	4	1.09 (0.94–1.26)	21.5	5	0.91 (0.79–1.04)	0.0	6	1.07 (0.89–1.27)	0.0
Postmenopausal		7	1.09 (0.96–1.25)	47.2	7	1.10 (0.97–1.26)	34.7	6	1.02 (0.80–1.31)	60.2	7	1.02 (0.91–1.14)	0.0
Regions:													
Asian		0	-	-	1	0.75 (0.48–1.15)	-	1	0.98 (0.10–9.53)	-	3	1.01 (0.82–1.25)	34.6
US		3	**1.09 (1.01–1.17)**	0.0	5	1.08 (0.99–1.17)	0.0	6	0.91 (0.80–1.04)	20.4	7	1.13 (0.94–1.35)	0.0
Europe		5	**1.34 (1.02–1.76)**	72.9	6	**1.20 (1.01–1.43)**	47.2	3	1.46 (0.78–2.74)	43.4	3	1.17 (0.73–1.86)	63.2
Duration of follow-up													
≤10 years		6	**1.19 (1.01–1.39)**	65.5	8	**1.12 (1.02–1.23)**	27.9	6	0.91 (0.79–1.03)	15.5	6	1.38 (0.91–2.10)	65.0
>10 years		2	**1.11 (1.00–1.23)**	0.0	4	1.05 (0.92–1.19)	4.5	4	1.11 (0.84–1.46)	32.2	7	1.01 (0.90–1.14)	0.0
Study type:													
Cohort		5	**1.11 (1.01–1.21)**	47.3	9	**1.10 (1.01–1.20)**	38.1	8	0.96 (0.84–1.11)	39.1	11	1.05 (0.94–1.18)	24.1
Nested case–control		2	**2.14 (1.34–3.43)**	0.0	2	1.11 (0.74–1.65)	0.0	2	1.35 (0.45–4.12)	42.6	2	1.87 (0.42–8.32)	76.3
Case–cohort		1	0.97 (0.76–1.23)	-	1	0.82 (0.27–2.49)	-	0	-	-	0	-	-
Study quality:													
Score > 7		5	1.06 (0.99–1.13)	0.0	6	**1.19 (1.01–1.13)**	0.0	3	1.04 (0.90–1.19)	0.0	6	1.08 (0.89–1.31)	43.3
Score ≤ 7		3	**1.56 (1.01–2.42)**	80.3	6	1.19 (0.97–1.45)	55.9	7	0.95 (0.74–1.21)	39.5	7	1.06 (0.88–1.30)	33.4
Unit													
g/day		5	**1.37 (1.01–1.86)**	72.1	5	1.21 (0.98–1.50)	60.3	4	1.08 (0.83–1.41)	39.5	4	1.11 (0.92–1.35)	37.8
Serving/day		2	1.17 (0.89–1.54)	11.3	6	1.08 (0.96–1.21)	0.0	5	0.97 (0.84–1.11)	44.7	9	1.04 (0.87–1.25)	36.3
Adjustment for confounders:													
Age at menarche	Yes	4	1.08 (0.97–1.21)	8.0	6	1.08 (0.96–1.22)	0.0	6	0.91 (0.80–1.04)	20.4	8	1.04 (0.91–1.19)	26.5
	No	4	**1.24 (1.03–1.51)**	77.1	6	1.13 (0.99–1.28)	59.2	4	1.26 (0.89–1.89)	15.3	5	1.16 (0.84–1.61)	51.1
Age at first birth	Yes	5	1.16 (0.93–1.43)	58.5	7	**1.12 (1.00–1.26)**	0.0	6	0.91 (0.80–1.04)	20.4	10	1.05 (0.90–1.22)	39.9
	No	3	1.14 (0.98–1.32)	69.4	5	1.09 (0.97–1.23)	53.4	4	1.26 (0.89–1.89)	15.3	3	1.15 (0.87–1.53)	19.0
Fat	Yes	0	-	-	1	1.13 (0.79–1.61)	-	1	0.90 (0.67–1.20)	-	2	1.76 (0.37–8.41)	81.6
	No	8	**1.36 (1.08–1.79)**	56.4	11	**1.10 (1.01–1.18)**	24.2	9	0.99 (0.84–1.16)	39.9	11	1.09 (0.94–1.25)	41.7
Smoking	Yes	7	**1.10 (1.01–1.19)**	38.8	7	**1.09 (1.02–1.16)**	10.6	5	0.97 (0.83–1.14)	54.2	5	0.98 (0.86–1.12)	0.0
	No	1	**2.19 (1.29–3.73)**	-	5	1.13 (0.90–1.38)	38.2	5	1.02 (0.69–1.52)	15.8	8	1.22 (0.94–1.58)	52.2
Alcohol	Yes	6	1.10 (0.97–1.26)	50.9	7	**1.09 (1.01–1.89)**	0.0	4	0.92 (0.73–1.17)	46.4	7	1.06 (0.94–1.19)	7.2
	No	2	1.34 (0.82–2.21)	83.6	5	1.14 (0.89–1.45)	52.7	6	1.01 (0.85–1.21)	27.3	6	1.13 (0.84–1.52)	56.6
BMI	Yes	7	**1.17 (1.01–1.36)**	63.0	10	**1.12 (1.03–1.22)**	11.3	6	1.01 (0.85–1.19)	30.4	10	1.03 (0.86–1.23)	39.0
	No	1	1.08 (0.98–1.09)	62	2	1.09 (0.67–1.76)	0.0	4	0.94 (0.74–1.20)	40.3	3	1.12 (0.99–1.27)	0.0
BMI + Alcohol	Yes	6	1.10 (0.97–1.26)	50.9	7	**1.09 (1.01–1.89)**	0.0	4	0.92 (0.73–1.17)	46.4	6	1.03 (0.86–1.25)	18.5
	No	2	1.34 (0.82–2.21)	83.6	5	1.14 (0.89–1.45)	52.7	6	1.01 (0.85–1.21)	27.3	7	1.09 (0.91–1.32)	46.3
Energy	Yes	6	1.09 (0.97–1.22)	45.9	7	**1.09 (1.01–1.17)**	9.4	5	1.05 (0.92–1.21)	0.0	6	1.08 (0.97–1.20)	0.0
	No	2	1.49 (0.79–2.83)	82.5	4	1.11 (0.93–1.32)	38.3	5	0.90 (0.71–1.13)	40.6	7	1.10 (0.83–1.46)	60.0
OC use	Yes	4	1.13 (0.93–1.38)	62.1	4	1.17 (0.93–1.47)	52.3	4	0.94 (0.77–1.16)	54.6	4	1.02 (0.95–1.09)	0
	No	4	1.15 (0.98–1.35)	63.6	8	**1.08 (1.02–1.15)**	0.0	6	1.03 (0.87–1.22)	4.7	9	1.07 (0.95–1.19)	57.7
Hormone therapy	Yes	4	1.35 (0.99–1.84)	74.7	9	**1.23 (1.01–1.49)**	54.1	6	0.96 (0.84–1.09)	43.6	7	1.05 (0.94–1.17)	7.4
	No	4	**1.13 (1.01–1.26)**	57.3	5	**1.07 (1.01–1.14)**	0.0	4	1.07 (0.90–1.27)	0.0	6	1.14 (0.81–1.59)	59.6
		**Egg**			**Total milk**			**Whole milk**			**Skim milk**		
		***n***	**RR (95% CI)**	***I*^2^**	***n***	**RR (95% CI)**	***I*^2^**	***n***	**RR (95% CI)**	***I*^2^**	***n***	**RR (95% CI)**	***I*^2^**
Overall		8	1.04 (0.94–1.15)	26.9	11	0.97 (0.93–1.01)	36.4	5	1.02 (0.92–1.13)	32.8	5	**0.96 (0.92–1.00)**	11.9
Menopausal status:													
Premenopausal		3	1.08 (0.91–1.27)	0.0	2	0.98 (0.92–1.04)	0.0	2	1.09 (0.90–1.32)	0.0	2	0.75 (0.38–1.48)	44.1
Postmenopausal		3	1.06 (0.97–1.17)	0.0	4	1.00 (0.96–1.03)	42.4	3	0.97 (0.89–1.06)	0.0	4	0.97 (0.94–1.01)	0.0
Regions:													
Asia		2	0.79 (0.46–1.35)	74.4	3	0.97 (0.77–1.23)	0.0	1	0.95 (0.35–2.54)	-	1	0.53 (0.24–1.20)	-
US		3	1.03 (0.84–1.22)	0.0	1	0.97 (0.94–1.01)	29.4	1	1.04 (0.94–1.16)	-	1	0.97 (0.94–1.01)	-
Europe		3	1.09 (1.00–1.19)	0.0	7	0.95 (0.87–1.03)	60.1	3	1.04 (0.90–1.19)	64.0	3	**0.94 (0.89–1.00)**	0.0
Duration of follow-up													
≤10 years		4	1.02 (0.81–1.28)	65.9	7	0.96 (0.91–1.01)	60.5	3	0.99 (0.92–1.06)	0.0	4	**0.96 (0.93–0.99)**	0.0
>10 years		4	1.02 (0.89–1.17)	0.0	4	1.01 (0.84–1.21)	0.0	2	1.30 (0.98–1.73)	0.0	1	0.53 (0.24–1.20)	-
Study type:													
Cohort		8	1.04 (0.94–1.15)	26.9	10	0.97 (0.92–1.01)	42.2	4	1.06 (0.96–1.17)	1.1	4	**0.96 (0.92–0.99)**	10.7
Nested case–control		0	-	-	0	-	-	0	-	-	0	-	-
Case–cohort		0	-	—	1	0.96 (0.86–1.07)	-	1	0.94 (0.85–1.05)	-	1	1.08 (0.87–1.33)	-
Study quality:													
Score > 7		2	1.07 (0.97–1.19)	0.0	7	0.98 (0.96–1.01)	0.0	4	0.99 (0.92–1.06)	0.0	4	0.96 (0.91–1.01)	31.1
Score ≤ 7		6	1.01 (0.87–1.18)	46.6	4	0.89 (0.69–1.17)	66.8	1	**1.34 (1.00–1.81)**	-	1	0.94 (0.85–1.05)	-
Unit													
g/day		5	**1.37 (1.01–1.86)**	72.1	7	0.99 (0.97–1.02)	0.0	4	1.03 (0.91–1.16)	46.1	3	0.96 (0.81–1.13)	45.7
Serving/day		2	1.17 (0.89–1.54)	11.3	4	0.88 (0.69–1.10)	73.0	1	0.94 (0.66–1.32)	-	2	**0.97 (0.93–1.00)**	0.0
Adjustment for confounders:													
Age at menarche	Yes	3	1.03 (0.84–1.22)	0.0	4	0.97 (0.94–1.00)	0.0	3	0.94 (0.85–1.04)	0.0	3	0.98 (0.86–1.12)	36.3
	No	5	1.03 (0.88–1.20)	55.0	7	0.94 (0.81–1.09)	56.8	2	1.14 (0.90–1.44)	59.9	2	**0.93 (0.88–0.99)**	0.0
Age at first birth	Yes	3	1.03 (0.84–1.22)	0.0	4	0.97 (0.94–1.00)	0.0	3	0.94 (0.85–1.04)	0.0	3	0.98 (0.86–1.12)	36.3
	No	5	1.03 (0.88–1.20)	55.0	7	0.94 (0.81–1.09)	56.8	2	1.14 (0.90–1.44)	59.9	2	**0.93 (0.88–0.99)**	0.0
Fat	Yes	0	-	-	0	-	-	0	-	-	0	-	-
	No	8	1.04 (0.94–1.15)	26.9	11	0.97 (0.93–1.01)	36.4	5	1.02 (0.92–1.13)	5	5	**0.96 (0.93–0.99)**	11.9
Smoking	Yes	3	1.06 (0.97–1.16)	0.0	4	1.00 (0.97–1.03)	0.0	3	0.99 (0.92–1.07)	0.0	3	0.96 (0.81–1.13)	45.7
	No	5	1.00 (0.79–1.25)	55.1	7	0.93 (0.85–1.02)	49.3	2	1.14 (0.80–1.61)	56.9	2	**0.97 (0.93–1.00)**	0.0
Alcohol	Yes	1	1.08 (0.84–1.38)	-	5	0.97 (0.94–1.00)	0.0	3	0.94 (0.85–1.04)	0.0	3	0.98 (0.86–1.12)	36.3
	No	7	1.03 (0.91–1.16)	37.1	6	0.92 (0.77–1.09)	62.6	2	1.14 (0.90–1.44)	59.9	2	**0.93 (0.88–0.99)**	0.0
BMI	Yes	3	**1.16 (1.00–1.36)**	0.0	6	0.93 (0.86–1.01)	57.9	3	0.94 (0.85–1.04)	0.0	4	0.97 (0.92–1.02)	13.9
	No	5	0.98 (0.84–1.13)	43.5	5	1.00 (0.97–1.03)	0.0	2	1.14 (0.90–1.44)	59.9	1	**0.93 (0.87–1.00)**	-
BMI + Alcohol	Yes	1	1.08 (0.84–1.38)	-	3	0.95 (0.89–1.02)	0.0	2	0.96 (0.91–1.02)	0.0	2	0.85 (0.44–1.64)	64.4
	No	7	1.03 (0.91–1.16)	37.1	8	0.97 (0.91–1.03)	53.2	3	1.08 (0.93–1.26)	30.3	3	**0.96 (0.93–0.99)**	0.0
Energy	Yes	2	0.83 (0.46–1.47)	85.2	6	1.00 (0.97–1.03)	0.0	3	0.99 (0.92–1.07)	0.0	2	**0.97 (0.93–1.00)**	0.0
	No	6	1.08 (0.97–1.21)	0.0	5	0.92 (0.83–1.03)	65.8	2	1.14 (0.80–1.61)	56.9	3	0.98 (0.86–1.12)	36.3
OC use	Yes	2	1.02 (0.85–1.21)	0.0	2	0.95 (0.89–1.02)	0.0	1	0.94 (0.85–1.05)	-	1	1.08 (0.87–1.33)	-
	No	6	1.04 (0.90–1.20)	44.0	9	0.97 (0.91–1.03)	46.7	4	1.06 (0.96–1.17)	1.1	4	0.96 (0.91–1.01)	31.1
Hormone therapy	Yes	2	1.02 (0.85–1.21)	0.0	3	0.97 (0.94–1.01)	0.0	1	0.95 (0.35–2.54)	-	2	0.82 (0.49–1.39)	53.8
	No	6	1.04 (0.90–1.20)	44.0	8	0.95 (0.88–1.02)	51.7	4	1.02 (0.91–1.15)	47.4	3	**0.94 (0.89–1.00)**	0.0

*n* denotes the number of studies; CI, confidence interval; RR, relative risk; OC, oral contraceptive; BMI: body mass index.

## Supplementary Materials

The following are available online at http://www.mdpi.com/2072-6643/8/11/730/s1, Table S1: Characteristics of included studies. Table S2: Quality assessment of included studies. Table S3: Subgroup analysis of dietary protein sources intake and risk of breast cancer, highest versus lowest intake.

## References

[1] Wolfe R.R. (2015). Update on protein intake: Importance of milk proteins for health status of the elderly. Nutr. Rev..

[2] Moughan P.J. (2012). Dietary protein for human health Preface. Br. J. Nutr..

[3] Lauber S.N., Ali S., Gooderham N.J. (2004). The cooked food derived carcinogen 2-amino-1-methyl-6-phenylimidazo[4,5-b] pyridine is a potent oestrogen: A mechanistic basis for its tissue-specific carcinogenicity. Carcinogenesis.

[4] Steck S.E., Gaudet M.M., Eng S.M., Britton J.A., Teitelbaum S.L., Neugut A.I., Santella R.M., Gammon M.D. (2007). Cooked meat and risk of breast cancer—Lifetime versus recent dietary intake. Epidemiology.

[5] Egeberg R., Olsen A., Autrup H., Christensen J., Stripp C., Tetens I., Overvad K., Tjønneland A. (2008). Meat consumption, N-acetyl transferase 1 and 2 polymorphism and risk of breast cancer, in Danish postmenopausal women. Eur. J. Cancer Prev..

[6] Taylor E.F., Burley V.J., Greenwood D.C., Cade J.E. (2007). Meat consumption and risk of breast cancer in the UK Women’s Cohort Study. Br. J. Cancer.

[7] Farvid M.S., Cho E., Chen W.Y., Eliassen A.H., Willett W.C. (2015). Adolescent meat intake and breast cancer risk. Int. J. Cancer.

[8] Farvid M.S., Cho E., Chen W.Y., Eliassen A.H., Willett W.C. (2014). Dietary protein sources in early adulthood and breast cancer incidence: Prospective cohort study. BMJ.

[9] Moher D., Liberati A., Tetzlaff J., Altman D.G., The PRISMA Group (2009). Preferred reporting items for systematic reviews and meta-analyses: The PRISMA statement. BMJ.

[10] Wells G.A., Shea B., O’connell D., Peterson J., Welch V., Losos M., Tugwell P. (2015). The Newcastle-Ottawa Scale (NOS) for Assessing Thequality of Nonrandomized Studies in Meta-Analysis.

[11] DerSimonian R., Laird N. (1986). Meta-analysis in clinical trials. Controll. Clin. Trials.

[12] Greenland S., Longnecker M.P. (1992). Methods for trend estimation from summarized dose-response data, with applications to meta-analysis. Am. J. Epidemiol..

[13] Food Standards Agency (2005). Food Portion Sizes.

[14] Norat T., Lukanova A., Ferrari P., Riboli E. (2002). Meat consumption and colorectal cancer risk: Dose-response meta-analysis of epidemiological studies. Int. J. Cancer.

[15] Keum N., Lee D.H., Marchand N., Oh H., Liu H., Aune D., Greenwood D.C., Giovannucci E.L. (2015). Egg intake and cancers of the breast, ovary and prostate: A dose-response meta-analysis of prospective observational studies. Br. J. Nutr..

[16] Bao Y., Hu F.B., Giovannucci E.L., Wolpin B.M., Stampfer M.J., Willett W.C., Fuchs C.S. (2013). Nut consumption and risk of pancreatic cancer in women. Br. J. Cancer.

[17] Ferrucci L.M., Cross A.J., Graubard B.I., Brinton L.A., McCarty C.A., Ziegler R.G., Ma X., Mayne S.T., Sinha R. (2009). Intake of meat, meat mutagens, and iron and the risk of breast cancer in the Prostate, Lung, Colorectal, and Ovarian Cancer Screening Trial. Br. J. Cancer.

[18] Cross A.J., Leitzmann M.F., Gail M.H., Hollenbeck A.R., Schatzkin A., Sinha R. (2007). A prospective study of red and processed meat intake in relation to cancer risk. PLoS Med..

[19] Higgins J.P., Thompson S.G., Deeks J.J., Altman D.G. (2003). Measuring inconsistency in meta-analyses. BMJ (Clin. Res. Ed.).

[20] Deeks J.J., Higgins J.P.T., Altman D.G., Higgins J., Green S. (2008). Cochranehandbook for Systematic Reviews of Interventions.

[21] Mills P.K., Beeson W.L., Phillips R.L., Fraser G.E. (1989). Dietary habits and breast cancer incidence among Seventh-day Adventists. Cancer.

[22] Ursin G., Bjelke E., Heuch I., Vollset S.E. (1990). Milk consumption and cancer incidence: A Norwegian prospective study. Br. J. Cancer.

[23] Vatten L.J., Solvoll K., Loken E.B. (1990). Frequency of meat and fish intake and risk of breast cancer in a prospective study of 14,500 Norwegian women. Int. J. Cancer.

[24] Toniolo P., Riboli E., Shore R.E., Pasternack B.S. (1994). Consumption of meat, animal products, protein, and fat and risk of breast cancer: A prospective cohort study in New York. Epidemiology.

[25] Gaard M., Tretli S., Loken E.B. (1995). Dietary fat and the risk of breast cancer: A prospective study of 25,892 Norwegian women. Int. J. Cancer.

[26] Byrne C., Ursin G., Ziegler R.G. (1996). A comparison of food habit and food frequency data as predictors of breast cancer in the NHANES I/NHEFS cohort. J. Nutr..

[27] Knekt P., Jarvinen R., Seppanen R., Pukkala E., Aromaa A. (1996). Intake of dairy products and the risk of breast cancer. Br. J. Cancer.

[28] Key T.J., Sharp G.B., Appleby P.N., Beral V., Goodman M.T., Soda M., Mabuchi K. (1999). Soya foods and breast cancer risk: A prospective study in Hiroshima and Nagasaki, Japan. Br. J. Cancer.

[29] Hjartaker A., Laake P., Lund E. (2001). Childhood and adult milk consumption and risk of premenopausal breast cancer in a cohort of 48,844 women—The Norwegian women and cancer study. Int. J. Cancer.

[30] Missmer S.A., Smith-Warner S.A., Spiegelman D., Yaun S.-S., Adami H.-O., Beeson W.L., van den Brandt P.A., Fraser G.E., Freudenheim J.L., Goldbohm R.A. (2002). Meat and dairy food consumption and breast cancer: A pooled analysis of cohort studies. Int. J. Epidemiol..

[31] Shin M.H., Holmes M.D., Hankinson S.E., Wu K., Colditz G.A., Willett W.C. (2002). Intake of dairy products, calcium, and vitamin d and risk of breast cancer. J. Natl. Cancer Inst..

[32] Voorrips L.E., Brants H.A.M., Kardinaal A.F.M., Hiddink G.J., van den Brandt P.A., Goldbohm R.A. (2002). Intake of conjugated linoleic acid, fat, and other fatty acids in relation to postmenopausal breast cancer: The Netherlands Cohort Study on Diet and Cancer. Am. J. Clin. Nutr..

[33] Gago-Dominguez M., Yuan J.M., Sun C.L., Lee H.P., Yu M.C. (2003). Opposing effects of dietary n-3 and n-6 fatty acids on mammary carcinogenesis: The Singapore Chinese Health Study. Br. J. Cancer.

[34] Holmes M.D., Colditz G.A., Hunter D.J., Hankinson S.E., Rosner B., Speizer F.E., Willett W.C. (2003). Meat, fish and egg intake and risk of breast cancer. Int. J. Cancer.

[35] Stripp C., Overvad K., Christensen J., Thomsen B.L., Olsen A., Moller S., Tjønneland A. (2003). Fish intake is positively associated with breast cancer incidence rate. J. Nutr..

[36] Yamamoto S., Sobue T., Kobayashi M., Sasaki S., Tsugane S., Japan Public Health Center-Based Prospective Study on Cancer Cardiovascular Diseases Group (2003). Soy, isoflavones, and breast cancer risk in Japan. J. Natl. Cancer Inst..

[37] Folsom A.R., Demissie Z. (2004). Fish intake, marine omega-3 fatty acids, and mortality in a cohort of postmenopausal women. Am. J. Epidemiol..

[38] Van der Hel O.L., Peeters P.H., Hein D.W., Doll M.A., Grobbee D.E., Ocke M., Bueno de Mesquita H.B. (2004). GSTM1 null genotype, red meat consumption and breast cancer risk (The Netherlands). Cancer Causes Control.

[39] Fung T.T., Hu F.B., Holmes M.D., Rosner B.A., Hunter D.J., Colditz G.A., Willett W.C. (2005). Dietary patterns and the risk of postmenopausal breast cancer. Int. J. Cancer.

[40] McCullough M.L., Rodriguez C., Diver W.R., Feigelson H.S., Stevens V.L., Thun M.J., Calle E.E. (2005). Dairy, calcium, and vitamin D intake and postmenopausal breast cancer risk in the Cancer Prevention Study II Nutrition Cohort. Cancer Epidemiol. Biomark. Prev..

[41] Shannon J., Ray R., Wu C., Nelson Z., Gao D.L., Li W., Hu W., Lampe J., Horner N., Satia J. (2005). Food and botanical groupings and risk of breast cancer: A case–control study in Shanghai, China. Cancer Epidemiol. Biomark. Prev..

[42] Wakai K., Tamakoshi K., Date C., Fukui M., Suzuki S., Lin Y.S., Niwa Y., Nishio K., Yatsuya H., Kondo T. (2005). Dietary intakes of fat and fatty acids and risk of breast cancer: A prospective study in Japan. Cancer Sci..

[43] Engeset D., Alsaker E., Lund E., Welch A., Khaw K.T., Clavel-Chapelon F., Thiebaut A., Chajes V., Key T.J., Allen N.E. (2006). Fish consumption and breast cancer risk. The European Prospective Investigation into Cancer and Nutrition (EPIC). Int. J. Cancer.

[44] Kesse-Guyot E., Bertrais S., Duperray B., Arnault N., Bar-Hen A., Galan P., Hercberg S. (2007). Dairy products, calcium and the risk of breast cancer: Results of the French SU.VI.MAX prospective study. Ann. Nutr. Metab..

[45] Nishio K., Niwa Y., Toyoshima H., Tamakoshi K., Kondo T., Yatsuya H., Yamamoto A., Suzuki S., Tokudome S., Lin Y. (2007). Consumption of soy foods and the risk of breast cancer: Findings from the Japan Collaborative Cohort (JACC) Study. Cancer Causes Control.

[46] Van der Pols J.C., Bain C., Gunnell D., Smith G.D., Frobisher C., Martin R.M. (2007). Childhood dairy intake and adult cancer risk: 65-y follow-up of the Boyd Orr cohort. Am. J. Clin. Nutr..

[47] Sonestedt E., Borgquist S., Ericson U., Gullberg B., Landberg G., Olsson H., Wirfält E. (2008). Plant foods and oestrogen receptor alpha- and beta-defined breast cancer: Observations from the Malmo Diet and Cancer cohort. Carcinogenesis.

[48] Larsson S.C., Bergkvist L., Wolk A. (2009). Long-term meat intake and risk of breast cancer by oestrogen and progesterone receptor status in a cohort of Swedish women. Eur. J. Cancer.

[49] Lee S.-A., Shu X.-O., Li H., Yang G., Cai H., Wen W., Ji B.T., Gao J., Gao Y.T., Zheng W. (2009). Adolescent and adult soy food intake and breast cancer risk: Results from the Shanghai Women’s Health Study. Am. J. Clin. Nutr..

[50] Pala V., Krogh V., Berrino F., Sieri S., Grioni S., Tjonneland A., Olsen A., Jakobsen M.U., Overvad K., Clavel-Chapelon F. (2009). Meat, eggs, dairy products, and risk of breast cancer in the European Prospective Investigation into Cancer and Nutrition (EPIC) cohort. Am. J. Clin. Nutr..

[51] Hjartaker A., Thoresen M., Engeset D., Lund E. (2010). Dairy consumption and calcium intake and risk of breast cancer in a prospective cohort: The Norwegian Women and Cancer study. Cancer Causes Control.

[52] Linos E., Willett W.C., Cho E., Frazier L. (2010). Adolescent diet in relation to breast cancer risk among premenopausal women. Cancer Epidemiol. Biomark. Prev..

[53] Wirfalt E., Li C., Manjer J., Ericson U., Sonestedt E., Borgquist S., Landberg G., Olsson H., Gullberg B. (2011). Food Sources of Fat and Sex Hormone Receptor Status of Invasive Breast Tumors in Women of the Malmo Diet and Cancer Cohort. Nutr. Cancer Int. J..

[54] Deschasaux M., Zelek L., Pouchieu C., His M., Hercberg S., Galan P., Latino-Martel P., Touvier M. (2013). Prospective Association between Dietary Fiber Intake and Breast Cancer Risk. PLoS ONE.

[55] Genkinger J.M., Makambi K.H., Palmer J.R., Rosenberg L., Adams-Campbell L.L. (2013). Consumption of dairy and meat in relation to breast cancer risk in the Black Women’s Health Study. Cancer Causes Control.

[56] Wada K., Nakamura K., Tamai Y., Tsuji M., Kawachi T., Hori A., Takeyama N., Tanabashi S., Matsushita S., Tokimitsu N. (2013). Soy isoflavone intake and breast cancer risk in Japan: From the Takayama study. Int. J. Cancer.

[57] Pouchieu C., Deschasaux M., Hercberg S., Druesne-Pecollo N., Latino-Martel P., Touvier M. (2014). Prospective association between red and processed meat intakes and breast cancer risk: Modulation by an antioxidant supplementation in the SU.VI.MAX randomized controlled trial. Int. J. Epidemiol..

[58] Wie G.-A., Cho Y.-A., Kang H.-H., Ryu K.-A., Yoo M.-K., Kim Y.-A., Jung K.W., Kim J., Lee J.H., Joung H. (2014). Red meat consumption is associated with an increased overall cancer risk: A prospective cohort study in Korea. Br. J. Nutr..

[59] Kiyabu G.Y., Inoue M., Saito E., Abe S.K., Sawada N., Ishihara J., Iwasaki M., Yamaji T., Shimazu T., Sasazuki S. (2015). Fish, *n*-3 polyunsaturated fatty acids and *n*-6 polyunsaturated fatty acids intake and breast cancer risk: The Japan Public Health Center-based prospective study. Int. J. Cancer.

[60] Gertig D.M., Hankinson S.E., Hough H., Spiegelman D., Colditz G.A., Willett W.C., Kelsey K.T., Hunter D.J. (1999). *N*-acetyl transferase 2 genotypes, meat intake and breast cancer risk. Int. J. Cancer.

[61] Daniel C.R., Cross A.J., Koebnick C., Sinha R. (2011). Trends in meat consumption in the USA. Public Health Nutr..

[62] Zheng W., Lee S.A. (2009). Well-done meat intake, heterocyclic amine exposure, and cancer risk. Nutr. Cancer.

[63] Farvid M.S., Cho E., Chen W.Y., Eliassen A.H., Willett W.C. (2014). Premenopausal dietary fat in relation to pre- and post-menopausal breast cancer. Breast Cancer Res. Treat..

[64] Fonseca-Nunes A., Jakszyn P., Agudo A. (2014). Iron and cancer risk—A systematic review and meta-analysis of the epidemiological evidence. Cancer Epidemiol. Biomark. Prev..

[65] Samraj A.N., Pearce O.M., Laubli H., Crittenden A.N., Bergfeld A.K., Banda K., Gregg C.J., Bingman A.E., Secrest P., Diaz S.L. (2015). A red meat-derived glycan promotes inflammation and cancer progression. Proc. Natl. Acad. Sci. USA.

[66] Andersson A.M., Skakkebaek N.E. (1999). Exposure to exogenous estrogens in food: Possible impact on human development and health. Eur. J. Endocrinol..

[67] Anderson L.N., Cotterchio M., Vieth R., Knight J.A. (2010). Vitamin D and calcium intakes and breast cancer risk in pre- and postmenopausal women. Am. J. Clin. Nutr..

[68] Cui Y., Rohan T.E. (2006). Vitamin D calcium, and breast cancer risk: A review. Cancer Epidemiol. Biomark. Prev..

[69] Sergeev I.N. (2012). Vitamin D and cellular Ca^2+^ signaling in breast cancer. Anticancer Res..

[70] Kelley N.S., Hubbard N.E., Erickson K.L. (2007). Conjugated linoleic acid isomers and cancer. J. Nutr..

[71] Key T.J., Appleby P.N., Cairns B.J., Luben R., Dahm C.C., Akbaraly T., Brunner E.J., Burley V., Cade J.E., Greenwood D.C. (2011). Dietary fat and breast cancer: Comparison of results from food diaries and food-frequency questionnaires in the UK Dietary Cohort Consortium. Am. J. Clin. Nutr..

[72] Kruk J., Marchlewicz M. (2013). Dietary Fat and Physical Activity in Relation to Breast Cancer among Polish Women. Asian Pac. J. Cancer Prev..

[73] Boeke C.E., Eliassen A.H., Chen W.Y., Cho E., Holmes M.D., Rosner B., Willett W.C., Tamimi R.M. (2014). Dietary fat intake in relation to lethal breast cancer in two large prospective cohort studies. Breast Cancer Res. Treat..

[74] Dong J.Y., Zhang L., He K., Qin L.Q. (2011). Dairy consumption and risk of breast cancer: A meta-analysis of prospective cohort studies. Breast Cancer Res. Treat..

[75] Taylor V.H., Misra M., Mukherjee S.D. (2009). Is red meat intake a risk factor for breast cancer among premenopausal women?. Breast Cancer Res. Treat..

[76] Zheng J.-S., Hu X.-J., Zhao Y.-M., Yang J., Li D. (2013). Intake of fish and marine n-3 polyunsaturated fatty acids and risk of breast cancer: Meta-analysis of data from 21 independent prospective cohort studies. BMJ.

[77] Alexander D.D., Morimoto L.M., Mink P.J., Cushing C.A. (2010). A review and meta-analysis of red and processed meat consumption and breast cancer. Nutr. Res. Rev..

[78] Guo J., Wei W., Zhan L. (2015). Red and processed meat intake and risk of breast cancer: A meta-analysis of prospective studies. Breast Cancer Res. Treat..

