# Supplementary material for: Dietary Protein Sources and Incidence of Breast Cancer: A Dose-Response Meta-Analysis of Prospective Studies
Source: Nutrients. 2016 Nov 17;8(11):730. doi: 10.3390/nu8110730 (PMC5133114; doi:10.3390/nu8110730)
Supplement: Supplementary file 1 [file nutrients-08-00730-s001.docx]

**SupplementaryMaterials: Dietary Protein Sources and Incidence of Breast Cancer: A Dose–Response Meta-Analysis of Prospective Studies**

Jing Wu, Rong Zeng, Junpeng Huang, Xufeng Li, Jiren Zhang, James Chung-Man Ho and Yanfang Zheng

**Table S1.** Characteristics of included studies.

| **Study Source** | **Study  Population** | **Case/ Participants** | **Follow-up  Duration** | **Baseline Age** | **Menopausal Status** | **Exposure  Details** | **Controlled Variables** |
| --- | --- | --- | --- | --- | --- | --- | --- |
| **Cohort** | | | | | | | |
| Mills et al./1989/US [1] | California Seventh–day Adventist cohort | 215/20,341 | 6 | 55.4 | Postmenopausal | Beef hamburger, beef steak,other beef/veal,beef index,fish, poultry, pork, whole milk, eggs | Age at entry, age at first live birth, age at menarche, menopausal status, history of benign breast disease, maternal history of breast cancer, educational attainment, and body mass index. |
| Vatten et al./1990/Norwegian [2] | Norwegian Cohort | 152/14,500 | 12 | 35–51 | Combined | Total meat, fish | Age. |
| Ursin et al./1990/Norwegian [3] | Norwegian Cohort | 29/2679 | 11.5 | 35–75 | Combined | Total milk | Age and region. |
| Gaard et al./1995/Norwegian [4] | Norwegian National Health Screening Services | 248/25,892 | 10.4 | 20–54 | Combined | Total meat, total milk, whole milk, eggs | Age. |
| Byrne et al./1996/US [5] | NHANES I/ NHEFS cohort | 53/6156 | 3.9 | 32–86 | Combined | Beef, whole milk | Age. |
| Knek  t et al./1996/Finland [6] | Mobile Health Clinic of the Social Institution | 88/4697 | 25 | 15–90 | Combined | Total milk, fermented milk | Age. |
| Key et al./1999/Japan [7] | Radiation Effects Research Foundation’s Life Span Study | 427/34,759 | 14 | 40–80 | Combined | Tofu, ham/sausage, fish(not dried), dried fish, milk, eggs | Attained age, calendar period, city, age at time of bombing, and radiation dose. |
| Hjartaker et al./2001/Norwegian [8] | Norwegian Cohort | 317/48,844 | 6.2 | 34–49 | Combined  (adult and childhood) | Total milk | Age, maternal history of breast cancer, age at menarche, number of children, age at first birth, current use of oral contraceptives, body mass index, years of education, physical activity, and alcohol consumption. |
| Missmer et al./2002/North America and Western Europe [9] | North America and Western Europe | 7379/35,1041 | 15 | 31–90 | Combined  (Premenopausal+  Postmenopausal) | Total meat, total red meat | Age at menarche, interaction between parity, age at first birth, oral contraceptive use, history of benign breast disease, family of breast cancer, smoking status, education, body mass index, height, alcohol intake, and total energy intake. |
| Shin et al./2002/US [10] | Nurses’ Health study cohort | 3482/88,691 | 16 | 30–55 | Premenopausal and  Postmenopausal | Whole milk, total milk, low–fat milk, milk intake during high school years, yogurt | Age, physical activity, history of benign breast disease, family history of breast cancer, height, weight change since age 18, body mass index at age 18, age at menarche, parity, age at first birth, alcohol intake, total energy intake, total fat intake, glycemic index, β-carotene intake, total active vitamin E intake, total calcium intake, total vitamin D intake, age at menopause, and postmenopausal hormone use. |
| Gago–Dominguez et al./2003/Singapore [11] | Singapore Chinese Health Study | 314/35,298 | 5.3 | 45–74 | Combined  (Premenopausal + Postmenopausal) | Fish | Age at baseline interview, year of recruitment, dialect group, education, daily alcohol drinker, family history of breast cancer, age when period became regular, and number of live births. |
| Holmes et al./2003/US [12] | Nurses’ Health study cohort | 4017/88,647 | 18 | 30–55 | Combined  (Premenopausal + Postmenopausal) | Fish, poultry, processed meat, total red meat, total meat, egg | Age, total energy intake, alcohol intake, parity and age at first birth categories, body mass index at age 18, weight change since age 18, height, family history of breast cancer, history of benign breast disease, age at menarche in years, menopausal status, age at menopausal and hormone replacement therapy, duration of menopause. |
| Stripp et al./2003/Danish [13] | Diet, Cancer, and Health Cohort | 424/23,693 | 4.8 | 50–65 | Postmenopausal | Fish | Parity, benign breast tumor, years of school, use of hormone replacement therapy, duration of hormone replacement therapy use, body mass index, and alcohol. |
| Yamamoto et al./2003/Japan [14] | JPHC cohort | 179/21,852 | 10 | 40–59 | Combined | Soy food | Area, age, age at menarche, number of pregnancies, menopausal status, age at first pregnancy, active and passive smoking, alcohol consumption, leisure-time physical activity, educational level, total energy and meat, fish, vegetable, and fruit consumption. |
| Folsom et al./2004/US [15] | Iowa women’s Health Study cohort | 1885/41,836 | 14 | 55–69 | Postmenopausal | Fish | Age, energy intake, educational level, physical activity level, alcohol consumption, smoking status, pack-years of cigarette smoking, age at first live birth, estrogen use, vitamin use, body mass index, waist/hip ratio, diabetes, hypertension, intake of whole grains, fruits, and vegetables, red meat, cholesterol, and saturated fat. |
| Fung et al./2005/US [16] | Nurses’ Health study cohort | 3026/71,058 | 16 | 30–55 | Postmenopausal | Soy foods | Age, smoking status, body mass index, multivitamin, energy intake, physical activity in metabolic equivalent hours, family history of breast cancer, history of benign breast disease, duration of menopause, age at menopause and use of hormone replacement therapy, age at menarche, parity and age at first birth, body mass index at age 18, weight change since age 18, height, and alcohol intake. |
| McCullough et al./2005/US [17] | Cancer Prevention Study Ⅱ Nutrition Cohort | 2855/68,567 | 7.8 | 50–74 | Postmenopausal | Total milk, low-fat milk | Age, energy, history of breast cyst, family history of breast cancer, height, weight gain since age 18, alcohol use, race, age at menopause, age at first birth and number of live births, education, mammography history, and hormone replacement therapy use. |
| Wakai et al./2005/Japan [18] | JACC cohort | 129/26,291 | 7.6 | 40–79 | Combined | Fish | Age, study area, educational level, family history of breast cancer, age at menarche, age at menopause, age at first birth, parity, use of exogenous female hormones, alcohol consumption, smoking, consumption of green leafy vegetables, daily walking, height, body mass index, and total energy intake. |
| Engeset et al./2006/European [19] | EPIC cohort | 4776/310,671 | 6.4 | 25–70 | Combined (Premenopausal+ Postmenopausal) | Fish | Centre, time of follow-up, energy intake from fat, energy intake from carbohydrates and protein, alcohol intake, height, weight, age at menarche, number of full-term pregnancies and age at first full-term pregnancies, current use of hormone replacement therapy, current use of oral contraceptives, and menopausal status. |
| Cross et al./2007/US [20] | NIH-AARP Diet and Health study | 5872/500,000 | 6.8 | 50–71 | Combined | Fresh red meat, processed meat | Age, education, marital status, family history of cancer, race, body mass index, smoking, frequency of vigorous physical activity, total energy intake, alcohol intake, and fruit and vegetable consumption. |
| Nishio et al./2007/Japan [21] | JACC cohort | 145/30,454 | 7.6 | 40–79 | Combined Postmenopausal | Tofu, boiled beans | Age, study area, family history of breast cancer, age at menopause, age at first birth, parity, use of exogenous female hormone, smoking, consumption of green leafy vegetables, walking time, body mass index, and total energy intake. |
| Taylor et al./2007/UK [22] | UK Women’s cohort | 678/33,725 | 8 | 35–69 | Combined (Premenopausal+ Postmenopausal) | Fresh red meat, processed red meat, poultry | Age, energy intake, menopausal status, body mass index, physical activity, smoking status, hormone replacement therapy use, oral contraceptive pill use, parity, total fruit and vegetable intake. |
| Van der Pols et al./2007/UK [23] | Boyd Orr Cohort | 97/2215 | 65 | 4–11 | Combined | Total milk | Age, sex, and energy, fruit, and calcium intakes. |
| Sonestedt et al./2008/Sweden [24] | Malmo Diet and Cancer cohort | 544/15,773 | 10.3 | 46–75 | Combined | Nuts | Season of data collection, diet interviewer, method version, age, total energy, weight, height, educational status, smoking habits, leisure time physical activity, hours of household activities, alcohol consumption, age at menopause, parity, and current use of menopausal hormone therapy. |
| Larsson et al./2009/Sweden [25] | Swedish Mammography cohort | 2952/61,433 | 17.4 |  | Combined | Total red meat, fresh red meat, processed meat | Age at entry, education, body mass index, height, parity and age at first birth, age at menarche, age at menopause, use of oral contraceptives, use of postmenopausal hormones, family history of breast cancer, intake of total energy, and alcohol intake. |
| Lee al./2009/China [26] | Shanghai Women’s health study | 594/73,225 | 7.4 | 40–70 | Combined (Premenopausal+ Postmenopausal) adolescence | Soy foods | Age, education, physical activity, age at first live birth, body mass index, season of recruitment, family history of breast cancer, total energy intake, and total fruit and vegetable intakes during adolescence. |
| Pala et al./2009/European [27] | EPIC cohort | 7119/31,9826 | 8.8 | 20–70 | Combined (Premenopausal+ Postmenopausal) | Red meat, poultry, processed meat, egg, all types of milk, whole milk, semi-skim milk, skim milk | Energy, height, weight, years of schooling, smoking, and menopause; stratified by center and age. |
| Hjartaker et al./2010/Norwegian [28] | Norwegian Women and Cancer Cohort | 1407/64,904 | 8.6 | 41.1–78.9 | Premenopausal and Postmenopausal | Total milk, yogurt | Age, energy intake, alcohol intake, height, weight increase since age 18, level of physical activity, years of education, maternal history of breast cancer, mammography practice, age at menarche, number of children and age at first birth, and use of oral contraceptives. |
| Linos et al./2010/US [29] | Nurses’ Health studyII cohort | 455/39,268 | 7.8 | 34–54 | Premenopausal adolescence | Total milk, low–fat milk, full–fat milk | Age, total energy intake, family history of breast cancer, history of benign breast disease, menopausal status, age at menarche, parity, age at first birth, weight gain since age 18 years, body mass index at age 18 years, current oral contraceptive, and adult alcohol use. |
| Wirfalt et al./2011/Sweden [30] | Malmo Diet and Cancer cohort | 544/15,773 | 10 | 45–73 | Combined | Yogurt, regular milk, low-fat milk, eggs, fatty meat, sausages, fatty fish, lean fish | Method version, season of data collection, age, and total energy. |
| Genkinger et al./2013/US [31] | Black Women’s Health Study | 1268/52,062 | 12 | 21–69 | Combined (Premenopausal+ Postmenopausal) | Total milk, yogurt, 2% milk, whole milk, skim milk, total meat, red meat, processed meat, white meat, fish | Energy intake, age at menarche, body mass index, family history of breast cancer, education, parity and age at first live birth, oral contraceptive use, menopausal status, age at menopause, menopausal hormone use, vigorous physical activity, smoking status, and alcohol intake. |
| Wada et al./2013/Japan [32] | Takayama cohort | 172/15,607 | 15.5 |  | Combined (Premenopausal+ Postmenopausal) | Soy foods | Age, body mass index, physical activity score, smoking status, alcohol consumption, education years, age at menarche, age at first delivery, menopausal status, parity number, and history of hormone replacement therapy. |
| Farvid et al./2014/US [33] | Nurses’ Health study IIcohort | 2830/88,803 | 20 | 26–45 | Combined | Red meat, poultry, fish, egg, legumes, nuts | Age, height, weight, family history of breast cancer, history of benign breast disease, smoking, race, age at menarche, parity, age at first birth, menopausal status, postmenopausal hormone use, age at menopause, and oral contraceptive use. |
| Wie et al./2014/Korea [34] | Cancer Screening Examination Cohort | 29/3622 | 7 |  | Combined | Total red meat | Age, energy intake, body mass index, physical activity, smoking, alcohol use, income, education, and marital status. |
| Farvid et al./2015/US [35] | Nurses’ Health study IIcohort | 1132/44,231 | 13 | 33–52 | Combined | Total red meat, fresh red meat, processed meat, poultry, fish, egg, legumes, nuts | Age, family history of breast cancer, history of benign breast disease, smoking, height, weight gain since age 18, body mass index at age 18 years, age at menarche, parity and age at first birth, oral contraceptive use, adult alcohol intake, postmenopausal hormone use, menopausal status, age at menopause. |
| Grace et al./2015/Japan [36] | JPHC cohort | 556/38,234 | 14.1 | 45–74 | Combined (Premenopausal+ Postmenopausal) | Fish | Area, body mass index, age at menarche, age at first birth, parity, menopausal age, menopausal status at baseline, use of exogenous female hormones, smoking status, alcohol intake, and total energy-adjusted intake of isoflavones. |
| **Nested case–control** | | | | | | | |
| Toniolo et al./1994/US [37] | New York City Cohort | 180/1009 | 6 | 35–65 | Combined | Total meat, poultry, fish | Energy. |
| Gertig et al./1999/US [38] | Nurses' Health study cohort | 466/932 | 18 | 30–55 | Combined | Total read meat, fish, chicken, processed meat | Age at menarche, parity, age at first birth, body mass index, family history of breast cancer in mother or sister, and history of benign breast disease. |
| Van der Hel et al./2004/Dutch [39] | Dutch prospective cohort | 229/551 | 10 | 20–59 | Combined | Total meat, fresh red meat, processed meat | Age, menopausal status, town, energy intake, smoking, alcohol, age at menarche, and body mass index. |
| Egeberg et al./2008/Danish [40] | Diet, Cancer, and Health Cohort | 378/24,697 | 4.2 | 50–64 | Postmenopausal | Total meat, read meat, fish, poultry, processed meat | Parity, age at first birth, education, duration of hormone replacement therapy use, intake of alcohol, and body mass index |
| **Case–cohort** | | | | | | | |
| Vorrips et al./2002/Netherlands [41] | Netherlands cohort | 941/2539 | 6.3 | 55–69 | Postmenopausal | Total milk, whole milk, skim milk, fermented whole milk, fermented skim milk, fresh red meat, beef, pork, processed meat | Age, history of benign breast disease, maternal breast cancer, breast cancer in one or more sisters, age at menarche, age at menopause, oral contraceptive use, parity, age at first childbirth, body mass index, education, alcohol use, current cigarette smoking, and energy intake. |
| **Randomized controlled trial** | | | | | | | |
| Shannon et al./2005/China [42] | Breast self-exam trial cohort | 378/1448 | 10 | 37–66 | Combined | Total meat, total read meat, fish, poultry, egg, soy food, total milk | Age, total energy intake, and breast-feeding. |
| Kesse-Guyot et al./2007/France [43] | SU.VI.MAX study | 92/3627 | 7.7 | 35–60 | Combined (Premenopausal+ Postmenopausal) | Total milk, yogurt | Educational level, parity, group of treatment, smoking status, overall physical activity, marital status, energy from fat, energy from other sources, alcohol intake, body mass index, family history of breast cancer in first degree, menopausal status and hormone replacement therapy use at baseline for the whole population, hormone replacement therapy use for menopausal women, and dietary energy-adjusted calcium intake. |
| Ferrucci et al./2009/US [44] | PLCO Cancer Screening Trial | 1205/52,158 | 5.5 | 55–74 | Combined | Total red meat, chicken, fish, processed meat | Age, race, education, study centre, randomization group, family history of breast cancer, age at menarche, age at menopause, age at first birth and number of live births, history of benign breast disease, menopausal hormone therapy use, body mass index, alcohol intake, total fat intake, and total energy intake. |
| Deschsauxet al./2013/France [45] | SU.VI.MAX study | 167/4684 | 12.6 | 55.8 | Combined | Soy foods | Age, intervention group, smoking status, educational level, physical activity, height, body mass index, number of dietary records, without-alcohol energy intake, alcohol intake, total fat intake, overall healthy dietary pattern, family history of breast cancer, menopausal status at baseline, use of hormonal treatment for menopause at baseline, and number of children. |
| Pouchieu et al./2014/France [46] | SU.VI.MAX study | 190/4684 | 11.3 | 55.8 | Combined | Fresh red meat, processed meat | Age, intervention group, number of dietary records, smoking status, educational level, physical activity, height, body mass index, family history of breast cancer, menopausal status at baseline, use of hormonal treatment for menopause at baseline, number of live births, without-alcohol energy intake, alcohol intake, total lipid intake. In addition, the red meat model is adjusted for processed meat intake and conversely (mutual adjustment). |

NHANES I:National Health and NutritionExamination SurveyI; NHEFS cohort: National Health Epidemiologic Follow-up Study cohort; JPHC: Japan Public Health Center-Based Prospective Study; JACC: Japan Collaborative Cohort; EPIC: European Prospective Investigation Into Cancer and Nutrition;NIH-AARP: National Institutes of Health -American Association for Retired PersonsDiet and Health Study; SU.VI.MAX: SUpplementation en VItamines et MInéraux AntioXydants; PLCO: Prostate, Lung, Colorectal, and Ovarian Cancer Screening Trial.

**Table S2.** Quality assessment of included studies.

| **Study** | **Selection** | **Comparability** | **Outcome/Exposure** | **Overall Quality** |
| --- | --- | --- | --- | --- |
| Mills et al./1989/US [1] | 3 | 2 | 3 | 8 |
| Vatten et al./1990/Norwegian [2] | 4 | 1 | 2 | 7 |
| Ursin et al./1990/Norwegian [3] | 3 | 1 | 2 | 6 |
| Gaard et al./1995/Norwegian [4] | 3 | 1 | 2 | 6 |
| Byrne et al./1996/US [5] | 4 | 1 | 1 | 6 |
| Knekt et al./1996/Finland [6] | 3 | 1 | 2 | 6 |
| Key et al./1999/Japan [7] | 3 | 1 | 3 | 7 |
| Hjartaker et al./2001/Norwegian [8] | 4 | 2 | 3 | 9 |
| Missmer et al./2002/North America and Western Europe [9] | 3 | 1 | 3 | 7 |
| Shin et al./2002/US [10] | 3 | 2 | 1 | 5 |
| Gago-Dominguez et al./2003/Singapore [11] | 4 | 1 | 3 | 8 |
| Holmes et al./2003/US [12] | 3 | 2 | 2 | 6 |
| Stripp et al./2003/Danish [13] | 4 | 1 | 2 | 7 |
| Yamamoto et al./2003/Japan [14] | 3 | 2 | 3 | 8 |
| Folsom et al./2004/US [15] | 4 | 2 | 2 | 8 |
| Fung et al./2005/US [16] | 3 | 2 | 1 | 6 |
| MoCullough et al./2005/US [17] | 4 | 2 | 2 | 8 |
| Wakai et al./2005/Japan [18] | 4 | 2 | 3 | 9 |
| Engeset et al./2006/European [19] | 4 | 1 | 3 | 8 |
| Cross et al./2007/US [20] | 4 | 2 | 2 | 8 |
| Nishio et al./2007/Japan [21] | 4 | 2 | 2 | 8 |
| Taylor et al./2007/UK [22] | 3 | 2 | 2 | 7 |
| Van der Pols et al./2007/UK [23] | 3 | 2 | 3 | 8 |
| Sonestedt et al./2008/Sweden [24] | 4 | 2 | 2 | 8 |
| Larsson et al./2009/Sweden [25] | 3 | 2 | 3 | 8 |
| Lee al./2009/China [26] | 3 | 2 | 3 | 8 |
| Pala et al./2009/European [27] | 4 | 2 | 3 | 9 |
| Hjartaker et al./2010/Norwegian [28] | 4 | 2 | 3 | 9 |
| Linos et al./2010/US [29] | 3 | 2 | 2 | 7 |
| Wirfalt et al./2011/Sweden [30] | 3 | 2 | 2 | 7 |
| Genkinger et al./2013/US [31] | 3 | 2 | 3 | 8 |
| Wada et al./2013/Japan [32] | 4 | 2 | 3 | 9 |
| Farvid et al./2014/US [33] | 3 | 2 | 2 | 7 |
| Wie et al./2014/Korea [34] | 3 | 1 | 3 | 7 |
| Farvid et al./2015/US [35] | 3 | 2 | 2 | 7 |
| Grace et al./2015/Japan [36] | 4 | 2 | 3 | 9 |
| Toniolo et al./1994/US [37] | 4 | 1 | 2 | 7 |
| Gertig et al./1999/US [38] | 3 | 2 | 2 | 7 |
| Van der Hel et al./2004/Dutch [39] | 3 | 2 | 3 | 8 |
| Egeberg et al./2008/Danish [40] | 3 | 2 | 2 | 7 |
| Vorrips et al./2002/Netherlands [41] | 4 | 2 | 3 | 9 |
| Shannon et al./2005/China [42] | 3 | 2 | 2 | 7 |
| Kesse–Guyot et al./2007/France [43] | 3 | 2 | 3 | 8 |
| Ferrucci et al./2009/US [44] | 4 | 2 | 2 | 8 |
| Deschsauxet al./2013/France [45] | 3 | 2 | 2 | 7 |
| Pouchieu et al./2014/France [46] | 3 | 2 | 2 | 7 |

**Table S3.** Subgroup analysis of dietary protein sources intake and risk of breast cancer, highest versus lowest intake.

|  | | **Total Red Meat** | | | **Fresh Red Meat** | | | **Processed Meat** | | | **Poultry** | | |
| --- | --- | --- | --- | --- | --- | --- | --- | --- | --- | --- | --- | --- | --- |
|  | | ***n*** | **RR (95% CI)** | ***I*^2^** | ***n*** | **RR (95% CI)** | ***I*^2^** | ***n*** | **RR (95% CI)** | ***I*^2^** | ***n*** | **RR (95% CI)** | ***I*^2^** |
| Overall | | 8 | 1.05 (0.95–1.16) | 63.1 | 12 | 1.07 (0.98–1.17) | 53.3 | 14 | **1.07 (1.01–1.14)** | 34.6 | 11 | 1.01 (0.93–1.11) | 58 |
| Menopausal status: | |  |  |  |  |  |  |  |  |  |  |  |  |
| Premenopausal | | 4 | 1.08 (0.92–1.27) | 44.7 | 4 | 1.06 (0.91–1.23) | 31.4 | 5 | 1.02 (0.88–1.17) | 36 | 6 | 0.95 (0.87–1.04) | 27.5 |
| Postmenopausal | | 4 | 0.98 (0.89–1.07) | 36.7 | 7 | 1.11 (0.95–1.30) | 43.9 | 7 | 1.09 (0.96–1.24) | 50 | 8 | 1.06 (0.93–1.21) | 65 |
| Regions: | |  |  |  |  |  |  |  |  |  |  |  |  |
| Asia | | 2 | 1.02 (0.51–2.05) | 28.9 | 0 | — | — | 1 | 0.78 (0.48–1.28) | — | 1 | 0.94 (0.59–1.49) | — |
| US | | 4 | 1.11 (0.94–1.31) | 68.8 | 5 | 1.02 (0.90–1.17) | 37.3 | 5 | 1.02 (0.95–1.10) | 24.1 | 7 | 0.99 (0.87–1.12) | 68.1 |
| Europe | | 1 | 0.98 (0.86–1.12) | — | 7 | 1.12 (0.98–1.30) | 64.5 | 8 | **1.13 (1.04–1.23)** | 16.9 | 3 | 1.09 (0.94–1.26) | 33.2 |
| North America and Western Europe | | 1 | 0.94 (0.87–1.02) | — | 0 | — | — | 0 | — | — | 0 | — | — |
| Duration of follow–up | |  |  |  |  |  |  |  |  |  |  |  |  |
| ≤10 years | | 3 | **1.21 (1.00–1.46)** | 0 | 8 | 1.10 (0.97–1.25) | 58.0 | 8 | **1.10 (1.02–1.19)** | 23.8 | 7 | 1.10 (0.97–1.26) | 46.5 |
| >10 years | | 5 | 1.03 (0.93–1.14) | 70.3 | 4 | 1.02 (0.89–1.18) | 44.4 | 6 | 1.04 (0.94–1.15) | 42.5 | 4 | 0.93 (0.82–106) | 66.2 |
| Study type: | |  |  |  |  |  |  |  |  |  |  |  |  |
| Cohort | | 8 | 1.05 (0.95–1.16) | 63.1 | 9 | 1.05 (0.95–1.15) | 54.6 | 11 | **1.07 (1.00–1.14)** | 37.6 | 9 | 1.00 (0.91–1.10) | 63.8 |
| Nested case–control | | 0 | — | — | 2 | **1.49 (1.10–2.02)** | 0 | 2 | 1.34 (0.92–1.96) | 18.8 | 2 | 1.23 (0.88–1.73) | 0 |
| Case–cohort | | 0 | — | — | 1 | 0.98 (0.73–1.33) | — | 1 | 0.93 (0.67–1.29) | — | 0 | — | — |
| Study quality: | |  |  |  |  |  |  |  |  |  |  |  |  |
| Score>7 | | 2 | 1.08 (0.87–1.35) | 69.8 | 7 | 1.02 (0.97–1.07) | 0 | 7 | **1.06 (1.01–1.12)** | 0 | 4 | 1.07 (0.93–1.23) | 63.6 |
| Score≤7 | | 6 | 1.05 (0.92–1.19) | 66.9 | 5 | 1.20 (0.93–1.54) | 62.7 | 7 | 1.15 (0.97–1.36) | 65.4 | 7 | 0.98 (0.86–1.11) | 56.6 |
| Adjustment for confounders: | |  |  |  |  |  |  |  |  |  |  |  |  |
| Age at menarche | Yes | 6 | 1.05 (0.95–1.16) | 71.2 | 6 | 1.01 (0.90–1.13) | 19.5 | 7 | 1.03 (0.96–1.10) | 6.6 | 6 | 0.98 (0.86–1.12) | 73.1 |
|  | No | 2 | 1.02 (0.51–2.05) | 28.9 | 6 | 1.12 (0.97–1.29) | 68.1 | 7 | **1.13 (1.02–1.26)** | 48 | 5 | 1.04 (0.97–1.12) | 0 |
| Age at first birth | Yes | 6 | 1.05 (0.95–1.16) | 71.2 | 6 | 1.05 (0.91–1.43) | 51.7 | 7 | 1.05 (0.96–1.15) | 40.6 | 7 | 1.00 (0.88–1.14) | 70.6 |
|  | No | 2 | 1.02 (0.51–2.05) | 28.9 | 6 | 1.09 (0.96–1.24) | 59.1 | 7 | **1.10 (1.00–1.20)** | 30.9 | 4 | 1.04 (0.96–1.110 | 0 |
| Fat | Yes | 1 | **1.23 (1.00–1.51)** | — | 0 | — | — | 1 | 1.12 (0.96–1.32) | — | 1 | 0.93 (0.77–1.13) | — |
|  | No | 7 | 1.03 (0.93–1.14) | 60.5 | 12 | 1.07 (0.98–1.17) | 53.3 | 13 | **1.07 (1.00–1.14)** | 38.7 | 10 | 1.03 (0.93–1.13) | 61.2 |
| Smoking | Yes | 5 | 1.11 (0.94–1.30) | 74.9 | 9 | **1.10 (1.02–1.20)** | 36 | 10 | **1.10 (1.03–1.19)** | 26.4 | 7 | 0.98 (0.88–1.09) | 60.4 |
|  | No | 3 | 0.96 (0.89–1.05) | 0 | 3 | 0.81 (0.59–1.12) | 34 | 4 | 1.00 (0.91–1.12) | 31.9 | 4 | 1.13 (0.89–1.43) | 58.6 |
| Alcohol | Yes | 6 | 1.00 (0.92–1.10) | 12.7 | 7 | 1.02 (0.94–1.09) | 13.4 | 8 | 1.03 (0.97–1.09) | 11.5 | 5 | 0.96(0.83–1.10) | 60.8 |
|  | No | 2 | **1.22 (1.07–1.40)** | 0 | 5 | 1.13 (0.86–1.48) | 72.1 | 6 | **1.15 (1.02–1.29)** | 34.3 | 6 | 1.07(0.94–1.23) | 60.8 |
| BMI | Yes | 6 | 1.00 (0.92–1.10) | 12.7 | 9 | 1.11 (0.99–1.25) | 56.3 | 10 | **1.09 (1.00–1.18)** | 47 | 7 | 1.02 (0.91–1.14) | 68.2 |
|  | No | 2 | **1.22 (1.07–1.40)** | 0 | 3 | 0.94 (0.72–1.23) | 62 | 4 | 1.08 (0.99–1.17) | 0 | 4 | 1.01 (0.85–1.20) | 34 |
| BMI+Alcohol | Yes | 6 | 1.00 (0.92–1.10) | 12.7 | 6 | 1.07(0.92–1.24) | 54.2 | 8 | 1.03 (0.97–1.09) | 11.5 | 5 | 0.96(0.83–1.10) | 60.8 |
|  | No | 2 | **1.22 (1.07–1.40)** | 0 | 6 | 1.08(0.95–1.23) | 58.5 | 6 | **1.15 (1.02–1.29)** | 34.3 | 6 | 1.07(0.94–1.23) | 60.8 |
| Energy | Yes | 6 | 0.99 (0.91–1.07) | 36.4 | 7 | 1.04 (0.96–1.14) | 50.6 | 10 | **1.05 (1.00–1.11)** | 25.5 | 7 | 1.02 (0.97–1.08) | 0 |
|  | No | 2 | **1.21 (1.07–1.35)** | 0 | 5 | 1.10 (0.82–1.48) | 58.3 | 4 | 1.21 (0.95–1.55) | 43 | 4 | 1.04 (0.77–1.41) | 83.9 |
| OC use | Yes | 4 | 1.06 (0.92–1.21) | 75.5 | 5 | 1.07 (0.91–1.26) | 66.4 | 5 | 1.10 (0.99–1.23) | 35.2 | 4 | 0.95 (0.79–1.14) | 73.5 |
|  | No | 4 | 1.06 (0.86–1.32) | 55.4 | 7 | 1.07 (0.95–1.21) | 47.6 | 9 | 1.06 (0.98–1.14) | 36.9 | 7 | 1.05 (0.95–1.15) | 39.1 |
| Hormone therapy | Yes | 5 | 1.09 (0.96–1.23) | 68.1 | 6 | 1.15 (0.97–1.37) | 70.2 | 9 | **1.10 (1.00–1.22)** | 53.6 | 7 | 0.98 (0.88–1.09) | 58.5 |
|  | No | 3 | 0.95 (0.87–1.03) | 0.5 | 6 | 1.03 (0.95–1.12) | 24.3 | 5 | **1.06 (1.00–1.13)** | 0 | 4 | 1.13 (0.90–1.43) | 58.3 |
|  | | **Fish** | | | **Total Milk** | | | **Whole Milk** | | | **Skim Milk** | | |
|  | | ***n*** | **RR (95% CI)** | ***I*^2^** | ***n*** | **RR (95% CI)** | ***I*^2^** | ***n*** | **RR (95% CI)** | ***I*^2^** | ***n*** | **RR (95% CI)** | ***I*^2^** |
| Overall | | 18 | 1.04 (0.97–1.12) | 47.9 | 18 | 0.92 (0.84–1.02) | 53.5 | 9 | 0.99 (0.87–1.12) | 37.4 | 8 | **0.93 (0.85–1.00)** | 40.1 |
| Menopausal status: | |  |  |  |  |  |  |  |  |  |  |  |  |
| Premenopausal | | 7 | 1.02 (0.93–1.12) | 0 | 6 | 0.95 (0.81–1.12) | 29.1 | 4 | 1.03 (0.91–1.17) | 0 | 4 | 0.86 (0.76–0.97) | 0 |
| Postmenopausal | | 11 | 1.02 (0.92–1.12) | 46.4 | 7 | 1.01 (0.92–1.10) | 33.3 | 5 | 1.02 (0.93–1.11) | 0 | 5 | 0.94 (0.88–1.01) | 0 |
| Regions: | |  |  |  |  |  |  |  |  |  |  |  |  |
| Asia | | 5 | 0.93 (0.73–1.19) | 61.5 | 2 | 0.94 (0.76–1.17) | 0 | 0 | — | — | 0 | — | — |
| US | | 8 | 1.03 (0.96–1.10) | 27.9 | 5 | 0.92 (0.82–1.03) | 29.4 | 6 | 0.92 (0.80–1.06) | 0 | 5 | 0.91 (0.82–1.01) | 24.8 |
| Europe | | 5 | 1.16 (0.96–1.40) | 58.2 | 11 | 1.01 (0.88–1.16) | 49.4 | 3 | 1.17 (0.82–1.68) | 75.4 | 3 | 0.95 (0.89–1.02) | 0 |
| Duration of follow-up | |  |  |  |  |  |  |  |  |  |  |  |  |
| ≤10 years | | 10 | 1.08 (0.92–1.27) | 66.9 | 9 | 1.00 (0.92–1.10) | 29.5 | 4 | 1.05 (0.97–1.14) | 0 | 4 | **0.93 (0.87–0.99)** | 0 |
| >10 years | | 8 | 1.01 (0.96–1.07) | 0 | 9 | 0.93 (0.78–1.10) | 53.2 | 5 | 0.99 (0.78–1.24) | 57.8 | 4 | 0.95 (0.82–1.08) | 43.2 |
| Study type: | |  |  |  |  |  |  |  |  |  |  |  |  |
| Cohort | | 16 | 1.04 (0.96–1.11) | 48.8 | 17 | 0.97 (0.89–1.06) | 46.4 | 8 | 1.00 (0.87–1.16) | 42.0 | 7 | **0.93 (0.87–0.99)** | 11.9 |
| Nested case–control | | 2 | 1.29 (0.84–1.98) | 35.6 | 0 | — | — | 0 | — | — | 0 | — | — |
| Case–cohort | | 0 | — | — | 1 | 0.91 (0.67–1.24) | — | 1 | 0.90 (0.66–1.22) | — | 1 | 1.04 (0.84–1.30) | — |
| Study quality: | |  |  |  |  |  |  |  |  |  |  |  |  |
| Score>7 | | 8 | 1.00 (0.89–1.14) | 62.3 | 9 | 0.99 (0.90–1.09) | 25.8 | 4 | 1.04 (0.96–1.12) | 0 | 4 | **0.92 (0.87–0.98)** | 0 |
| Score≤7 | | 10 | 1.07 (0.98–1.17) | 35.4 | 9 | 0.94 (0.80–1.10) | 57.9 | 5 | 1.06 (0.75–1.49) | 61.9 | 4 | 0.97 (0.83–1.13) | 34.1 |
| Adjustment for confounders: | |  |  |  |  |  |  |  |  |  |  |  |  |
| Age at menarche | Yes | 10 | 1.02 (0.94–1.10) | 51 | 9 | 0.93 (0.84–1.03) | 31.2 | 6 | 0.92 (0.81–1.05) | 0 | 6 | 0.93 (0.84–1.02) | 21.9 |
|  | No | 8 | 1.13 (0.96–1.32) | 47.3 | 9 | 1.02 (0.88–1.18) | 45 | 3 | 1.31 (0.58–2.93) | 74.7 | 2 | 0.95 (0.86–1.06) | 21.8 |
| Age at first birth | Yes | 11 | 1.04 (0.96–1.12) | 46.9 | 9 | 0.93 (0.84–1.03) | 31.2 | 6 | 0.92 (0.81–1.05) | 0 | 6 | 0.93 (0.84–1.02) | 21.9 |
|  | No | 7 | 1.07 (0.89–1.30) | 55.7 | 9 | 1.02 (0.88–1.18) | 45 | 3 | 1.31 (0.58–2.93) | 74.7 | 2 | 0.95 (0.86–1.06) | 21.8 |
| Fat | Yes | 1 | 1.08 (0.89–1.31) | — | 2 | 1.62 (0.94–2.81) | 0.0 | 2 | 0.87 (0.71–1.06) | 0.0 | 2 | 0.92 (0.69–1.23) | 73.3 |
|  | No | 17 | 1.04 (0.97–1.13) | 50.6 | 16 | 0.94 (0.84–1.04) | 49.2 | 7 | 1.03 (0.89–1.21) | 39.5 | 6 | **0.93 (0.88–0.99)** | 0.0 |
| Smoking | Yes | 8 | 1.00 (0.91–1.09) | 31.7 | 4 | 1.04 (0.97–1.13) | 0 | 3 | 1.04 (0.96–1.13) | 0 | 3 | **0.93 (0.87–1.00)** | 0 |
|  | No | 10 | 1.10 (0.98–1.23) | 54.1 | 14 | 0.94 (0.84–1.06) | 50.2 | 6 | 1.01 (0.78–1.31) | 52.4 | 5 | **0.94 (0.84–1.00)** | 34.8 |
| Alcohol | Yes | 9 | 1.00 (0.91–1.10) | 53.5 | 10 | 0.94 (0.85–1.04) | 29 | 5 | 0.92 (0.80–1.05) | 0 | 6 | 0.93 (0.84–1.02) | 21.9 |
|  | No | 9 | 1.12 (0.99–1.27) | 45.6 | 8 | 1.01 (0.86–1.18) | 50 | 4 | 1.15 (0.80–1.65) | 64.3 | 2 | 0.95 (0.86–1.06) | 21.8 |
| BMI | Yes | 10 | 1.07 (0.96–1.20) | 60.6 | 6 | 0.92 (0.76–1.10) | 48.2 | 5 | 0.93 (0.81–1.06) | 0 | 5 | 0.95(0.84–1.07) | 27.2 |
|  | No | 8 | 1.01 (0.92–1.11) | 23.9 | 12 | 0.99 (0.90–1.10) | 42.9 | 4 | 1.12 (0.79–1.59) | 66.9 | 3 | **0.93(0.87–0.99)** | 0.0 |
| Alcohol + BMI | Yes | 9 | 1.00 (0.91–1.10) | 53.5 | 6 | 0.92 (0.76–1.10) | 48.2 | 5 | 0.93 (0.81–1.06) | 0 | 5 | 0.95(0.84–1.07) | 27.2 |
|  | No | 9 | 1.12 (0.99–1.27) | 45.6 | 12 | 0.99 (0.90–1.10) | 42.9 | 4 | 1.12 (0.79–1.59) | 66.9 | 3 | **0.93(0.87–0.99)** | 0.0 |
| Energy | Yes | 10 | 1.02 (0.96–1.08) | 6.6 | 13 | 1.00 (0.94–1.06) | 11.6 | 6 | 1.02 (0.95–1.09) | 0 | 7 | 0.95 (0.89–1.02) | 12.5 |
|  | No | 8 | 1.14 (0.97–1.34) | 68.8 | 5 | 0.84 (0.52–1.36) | 73.1 | 3 | 1.24 (0.50–3.06) | 76.0 | 1 | 0.88 (0.76–1.02) | — |
| OC use | Yes | 4 | 1.02 (0.95–1.09) | 0 | 6 | 0.99 (0.86–1.13) | 11.4 | 3 | 0.96 (0.80–1.16) | 0 | 3 | 0.94 (0.81–1.09) | 0 |
|  | No | 14 | 1.07 (0.95–1.19) | 57.7 | 12 | 0.96 (0.86–1.08) | 55.2 | 6 | 1.01 (0.82–1.23) | 57.4 | 5 | 0.94 (0.87–1.02) | 34.4 |
| Hormone therapy | Yes | 11 | 1.03 (0.96–1.11) | 42.5 | 5 | 0.92 (0.81–1.06) | 40.2 | 3 | 0.90 (0.77–1.06) | 0 | 4 | **0.91 (0.80–1.00)** | 42.5 |
|  | No | 7 | 1.08 (0.88–1.32) | 59.6 | 13 | 0.99 (0.89–1.11) | 40.3 | 6 | 1.06 (0.87–1.29) | 47.4 | 4 | 0.95 (0.89–1.02) | 0 |

*n* denotes the number of studies; CI: confidence interval; RR:relative risk; OC:oral contraceptive; BMI: body mass index;we did not conduct the meta–regression and subgroup analysis due to no evidence of heterogeneity in the summary analysis (*I*_soyfood_^2^=0.0%, *I*_yogurt_^2^=0.0%, *I*_egg_^2^=6.7%), and as only three cohort studies investigated the association between nut intake and risk of breast cancer, we also did not conduct the meta–regression and subgroup analysis for nut.

References

1. Mills, P.K.; Beeson, W.L.; Phillips, R.L.; Fraser, G.E. Dietary habits and breast cancer incidence among Seventh-day Adventists. *Cancer* **1989**, *64*, 582–590.
2. Vatten, L.J, Solvoll, K.; Loken, E.B. Frequency of meat and fish intake and risk of breast cancer in a prospective study of 14,500 Norwegian women*. Int. J. Cancer***1990**, *46*, 12–15.
3. Ursin, G.; Bjelke, E.; Heuch, I.; Vollset, S.E. Milk consumption and cancer incidence: A Norwegian prospective study. *Br. J. Cancer* **1990**, *61*, 454–459.
4. Gaard, M.; Tretli, S.; Loken, E.B. Dietary fat and the risk of breast cancer: A prospective study of 25,892 Norwegian women. *Int. J. Cancer* **1995**, *63*, 13–17.
5. Byrne, C.; Ursin, G.; Ziegler, R.G. A comparison of food habit and food frequency data as predictors of breast cancer in the NHANES I/NHEFS cohort*. J. Nutr.* **1996**, *126*, 2757–2764.
6. Knekt, P.; Jarvinen, R.; Seppanen, R.; Pukkala, E.; Aromaa, A. Intake of dairy products and the risk of breast cancer. *Br. J. Cancer* **1996**, *73*, 687–691.
7. Key, T.J.; Sharp, G.B.; Appleby, P.N.; Beral, V.; Goodman, M.T.; Soda, M.; Mabuchi, K. Soya foods and breast cancer risk: A prospective study in Hiroshima and Nagasaki, Japan. *Br. J. Cancer* **1999**, *81*, 1248–1256, doi:10.1038/sj.bjc.6690837.
8. Hjartaker, A.; Laake, P.; Lund, E. Childhood and adult milk consumption and risk of premenopausal breast cancer in a cohort of 48,844 women—The Norwegian women and cancer study. *Int. J. Cancer* **2001**, *93*, 888–893.
9. Missmer, S.A.; Smith-Warner, S.A.; Spiegelman, D.; Yaun, S.-S.; Adami, H.-O.; Beeson, W.L.; van den Brandt, P.A.; Fraser, G.E.; Freudenheim, J.L.; Goldbohm, R.A.; et al. Meat and dairy food consumption and breast cancer: A pooled analysis of cohort studies. *Int. J. Epidemiol.* **2002**, *31*, 78–85.
10. Shin, M.H.; Holmes, M.D.; Hankinson, S.E.; Wu, K.; Colditz, G.A.; Willett, W.C. Intake of dairy products, calcium, and vitamin d and risk of breast cancer. *J. Natl. Cancer Inst.* **2002**, *94*, 1301–1311.
11. Gago-Dominguez, M.; Yuan, J.M.; Sun, C.L.; Lee, H.P.; Yu, M.C. Opposing effects of dietary n-3 and n-6 fatty acids on mammary carcinogenesis: The Singapore Chinese Health Study. *Br. J. Cancer* **2003**, *89*, 1686–1692, doi:10.1038/sj.bjc.6601340.
12. Holmes, M.D.; Colditz, G.A.; Hunter, D.J.; Hankinson, S.E.; Rosner, B.; Speizer, F.E.; Willett, W.C. Meat, fish and egg intake and risk of breast cancer. *Int. J. Cancer* **2003**, *104*, 221–227.
13. Stripp, C.; Overvad, K.; Christensen, J.; Thomsen, B.L.; Olsen, A.; Moller, S.; Tjønneland, A. Fish intake is positively associated with breast cancer incidence rate. *J. Nutr.* **2003**, *133*, 3664–3669.
14. Yamamoto, S.; Sobue, T.; Kobayashi, M.; Sasaki, S.; Tsugane, S.; Japan Public Health Center-Based Prospective Study on Cancer Cardiovascular Diseases Group. Soy, isoflavones, and breast cancer risk in Japan. *J. Natl. Cancer Inst.* **2003**, *95*, 906–913.
15. Folsom, A.R.; Demissie, Z. Fish intake, marine omega-3 fatty acids, and mortality in a cohort of postmenopausal women*. Am. J. Epidemiol.* **2004**, *160*, 1005–1010, doi:10.1093/aje/kwh307.
16. Fung, T.T.; Hu, F.B.; Holmes, M.D.; Rosner, B.A.; Hunter, D.J.; Colditz, G.A.; Willett, W.C. Dietary patterns and the risk of postmenopausal breast cancer. *Int. J. Cancer* **2005**, *116*, 116–121, doi:10.1002/ijc.20999.
17. McCullough, M.L.; Rodriguez, C.; Diver, W.R.; Feigelson, H.S.; Stevens, V.L.; Thun, M.J., Calle, E.E. Dairy, calcium, and vitamin D intake and postmenopausal breast cancer risk in the Cancer Prevention Study II Nutrition Cohort. *Cancer Epidemiol. Biomark. Prev.* **2005**, *14*, 2898–2904.
18. Wakai, K.; Tamakoshi, K.; Date, C.; Fukui, M.; Suzuki, S.; Lin, Y.S.; Niwa, Y.; Nishio, K.; Yatsuya, H.; Kondo, T.; et al. Dietary intakes of fat and fatty acids and risk of breast cancer: A prospective study in Japan. *Cancer Sci.* **2005**, *96*, 590–599.
19. Engeset, D.; Alsaker, E.; Lund, E.; Welch, A.; Khaw, K.T.; Clavel-Chapelon, F.; Thiébaut, A.; Chajès, V.; Key, T.J.; Allen, N.E.; et al. Fish consumption and breast cancer risk. The European Prospective Investigation into Cancer and Nutrition (EPIC). *Int. J. Cancer* **2006**, *119*, 175–182.
20. Cross, A.J.; Leitzmann, M.F.; Gail, M.H.; Hollenbeck, A.R.; Schatzkin, A.; Sinha, R. A prospective study of red and processed meat intake in relation to cancer risk. *PLoS Med.* **2007**, *4*, e325.
21. Nishio, K.; Niwa, Y.; Toyoshima, H.; Tamakoshi, K.; Kondo, T.; Yatsuya, H.; Yamamoto, A.; Suzuki, S.; Tokudome, S.; Lin, Y.; et al. Consumption of soy foods and the risk of breast cancer: Findings from the Japan Collaborative Cohort (JACC) Study. *Cancer Causes Control* **2007**, *18*, 801–808.
22. Taylor, E.F.; Burley, V.J.; Greenwood, D.C.; Cade, J.E. Meat consumption and risk of breast cancer in the UK Women’s Cohort Study. *Br. J. Cancer* **2007**, *96*, 1139–1146, doi:10.1038/sj.bjc.6603689.
23. Van der Pols, J.C.; Bain, C.; Gunnell, D.; Smith, G.D.; Frobisher, C.; Martin, R.M. Childhood dairy intake and adult cancer risk: 65-y follow-up of the Boyd Orr cohort. *Am. J. Clin. Nutr.* **2007**, *86*, 1722–1729.
24. Sonestedt, E.; Borgquist, S.; Ericson, U.; Gullberg, B.; Landberg, G.; Olsson, H.; Wirfält, E. Plant foods and oestrogen receptor alpha- and beta-defined breast cancer: Observations from the Malmo Diet and Cancer cohort. *Carcinogenesis* **2008**, *29*, 2203–2209.
25. Larsson, S.C.; Bergkvist, L.; Wolk, A. Long-term meat intake and risk of breast cancer by oestrogen and progesterone receptor status in a cohort of Swedish women. *Eur. J. Cancer* **2009**, *45*, 3042–3046, doi:10.1016/j.ejca.2009.04.035.
26. Lee, S.-A.; Shu, X.-O.; Li, H.; Yang, G.; Cai, H.; Wen, W.; Ji, B.T.; Gao, J.; Gao, Y.T.; Zheng, W. Adolescent and adult soy food intake and breast cancer risk: Results from the Shanghai Women’s Health Study. *Am. J. Clin. Nutr.* **2009**, *89*, 1920–1926, doi:10.3945/ajcn.2008.27361.
27. Pala, V.; Krogh, V.; Berrino, F.; Sieri, S.; Grioni, S.; Tjonneland, A.; Olsen, A.; Jakobsen, M.U.; Overvad, K.; Clavel-Chapelon, F.; et al. Meat, eggs, dairy products, and risk of breast cancer in the European Prospective Investigation into Cancer and Nutrition (EPIC) cohort. *Am. J. Clin. Nutr.* **2009**, *90*, 602–612, doi:10.3945/ajcn.2008.27173.
28. Hjartaker, A.; Thoresen, M.; Engeset, D.; Lund, E. Dairy consumption and calcium intake and risk of breast cancer in a prospective cohort: The Norwegian Women and Cancer study*. Cancer Causes Control* **2010**, *21*, 1875–1885, doi:10.1007/s10552-010-9615-5.
29. Linos, E.; Willett, W.C.; Cho, E.; Frazier, L. Adolescent diet in relation to breast cancer risk among premenopausal women. *Cancer Epidemiol. Biomark. Prev.* **2010**, *19*, 689–696, doi:10.1158/1055-9965.EPI-09-0802.
30. Wirfalt, E.; Li, C.; Manjer, J.; Ericson, U.; Sonestedt, E.; Borgquist, S.; Landberg, G.; Olsson, H.; Gullberg, B. Food Sources of Fat and Sex Hormone Receptor Status of Invasive Breast Tumors in Women of the Malmo Diet and Cancer Cohort. *Nutr. Cancer Int. J.* **2011**, *63*, 722–733, doi:10.1080/01635581.2011.570897.
31. Genkinger, J.M.; Makambi, K.H.; Palmer, J.R.; Rosenberg, L.; Adams-Campbell, L.L. Consumption of dairy and meat in relation to breast cancer risk in the Black Women’s Health Study. *Cancer Causes Control* **2013**, *24*, 675–684, doi:10.1007/s10552-013-0146-8.
32. Wada, K.; Nakamura, K.; Tamai, Y.; Tsuji, M.; Kawachi, T.; Hori, A.; Takeyama, N.; Tanabashi, S.; Matsushita, S.; Tokimitsu, N.; et al. Soy isoflavone intake and breast cancer risk in Japan: From the Takayama study. *Int. J. Cancer* **2013**, *133*, 952–960, doi:10.1002/ijc.28088.
33. Farvid, M.S.; Cho, E.; Chen, W.Y.; Eliassen, A.H.; Willett, W.C. Dietary protein sources in early adulthood and breast cancer incidence: Prospective cohort study. *BMJ* **2014**, *348*, doi:10.1136/bmj.g3437.
34. Wie, G.-A.; Cho, Y.-A.; Kang, H.-H.; Ryu, K.-A.; Yoo, M.-K.; Kim, Y.-A.; Jung, K.W.; Kim, J.; Lee, J.H.; Joung, H. Red meat consumption is associated with an increased overall cancer risk: A prospective cohort study in Korea. *Br. J. Nutr.* **014**, *112*, 238–247, doi:10.1017/S0007114514000683.
35. Farvid, M.S.; Cho, E.; Chen, W.Y.; Eliassen, A.H.; Willett, W.C. Adolescent meat intake and breast cancer risk. *Int. J. Cancer* **2015**, *136*, 1909–1920, doi:10.1002/ijc.29218.
36. Kiyabu, G.Y.; Inoue, M.; Saito, E.; Abe, S.K.; Sawada, N.; Ishihara, J.; Iwasaki, M.; Yamaji, T.; Shimazu, T.; Sasazuki, S. et al. Fish, n-3 polyunsaturated fatty acids and n-6 polyunsaturated fatty acids intake and breast cancer risk: The Japan Public Health Center-based prospective study. *Int. J. Cancer* **2015**, *137*, 2915–2926.
37. Toniolo, P.; Riboli, E.; Shore, R.E.; Pasternack, B.S. Consumption of meat, animal products, protein, and fat and risk of breast cancer: A prospective cohort study in New York. *Epidemiology* **1994**, *5*, 391–397.
38. Gertig, D.M.; Hankinson, S.E.; Hough, H.; Spiegelman, D.; Colditz, G.A.; Willett, W.C.; Kelsey, K.T.; Hunter, D.J. N-acetyl transferase 2 genotypes, meat intake and breast cancer risk. *Int. J. Cancer* **1999**, *80*, 13–17.
39. Van der Hel, O.L.; Peeters, P.H.; Hein, D.W.; Doll, M.A.; Grobbee, D.E.; Ocke, M.; Bueno de Mesquita, H.B. GSTM1 null genotype, red meat consumption and breast cancer risk (The Netherlands). *Cancer Causes Control* **2004**, *15*, 295–303.
40. Egeberg, R.; Olsen, A.; Autrup, H.; Christensen, J.; Stripp, C.; Tetens, I.; Overvad, K.; Tjønneland, A. Meat consumption, *N*-acetyl transferase 1 and 2 polymorphism and risk of breast cancer, in Danish postmenopausal women. *Eur. J. Cancer Prev.* **2008**, *17*, 39–47, doi:10.1097/CEJ.0b013e32809b4cdd.
41. Voorrips, L.E.; Brants, H.A.M.; Kardinaal, A.F.M.; Hiddink, G.J.; van den Brandt, P.A.; Goldbohm, R.A. Intake of conjugated linoleic acid, fat, and other fatty acids in relation to postmenopausal breast cancer: The Netherlands Cohort Study on Diet and Cancer. *Am. J. Clin. Nutr.* **2002**, *76*, 873–882.
42. Shannon, J.; Ray, R.; Wu, C.; Nelson, Z.; Gao, D.L.; Li, W.; Hu, W.; Lampe, J.; Horner, N.; Satia, J.; et al. Food and botanical groupings and risk of breast cancer: A case–control study in Shanghai, China. *Cancer Epidemiol. Biomark. Prev.* **2005**, *14*, 81–90.
43. Kesse-Guyot, E.; Bertrais, S.; Duperray, B.; Arnault, N.; Bar-Hen, A.; Galan, P.; Hercberg S. Dairy products, calcium and the risk of breast cancer: Results of the French SU.VI.MAX prospective study. *Ann. Nutr. Metab.* **2007**, *51*, 139–145.
44. Ferrucci, L.M.; Cross, A.J.; Graubard, B.I.; Brinton, L.A.; McCarty, C.A.; Ziegler, R.G.; Ma, X.; Mayne, S.T.; Sinha, R. Intake of meat, meat mutagens, and iron and the risk of breast cancer in the Prostate, Lung, Colorectal, and Ovarian Cancer Screening Trial. *Br. J. Cancer* **2009**, *101*, 178–184.
45. Deschasaux, M.; Zelek, L.; Pouchieu, C.; His, M.; Hercberg, S.; Galan, P.; Latino-Martel, P.; Touvier, M. Prospective Association between Dietary Fiber Intake and Breast Cancer Risk. *PLoS ONE* **2013**, *8*, e79718.
46. Pouchieu, C.; Deschasaux, M.; Hercberg, S.; Druesne-Pecollo, N.; Latino-Martel, P.; Touvier, M. Prospective association between red and processed meat intakes and breast cancer risk: Modulation by an antioxidant supplementation in the SU.VI.MAX randomized controlled trial. *Int. J. Epidemiol.* **2014**, *43*, 1583–1592, doi:10.1093/ije/dyu134.

© 2016 by the authors. Submitted for possible open access publication under the terms and conditions of the Creative Commons Attribution (CC-BY) license (http://creativecommons.org/licenses/by/4.0/).
